# Identifying promoters to enhance heterologous gene expression in recombinant *Saccharomyces cerevisiae* strains cultivated on non-native substrates

**DOI:** 10.1007/s00253-025-13563-6

**Published:** 2025-07-26

**Authors:** Jordan Fortuin, Riaan den Haan

**Affiliations:** Institute for Microbial Biotechnology and Metagenomics, Department of Biotechnology, University of the Western Cape, Bellville, South Africa

**Keywords:** Promoter, Heterologous expression, Lignocellulosic biomass, Consolidated bioprocessing, Xylan, *Saccharomyces cerevisiae*

## Abstract

**Abstract:**

Efficient bioconversion of lignocellulosic biomass (LCB) to ethanol by *Saccharomyces cerevisiae* requires its engineering to express heterologous enzymes at titres high enough to make significant impacts on industrial consolidated bioprocessing (CBP). Promoters are required for this purpose, but are reportedly influenced by various environmental factors as well as the protein specific nature of expression, warranting the need for assessment under the conditions for which they are intended. Heterologous xylosidase- and xylanase-encoding genes (*xln43_SED1* and *xyn2*) were individually cloned under transcriptional control of the *SED1*_P_ and *TDH3*_P_ promoters, and *DIT1*_T_ terminator, and integrated into the genome of an a *S. cerevisiae* strain engineered for xylose utilization. Enzymatic assays were used to quantify the performance of the promoters when strains were cultivated on glucose (aerobically and micro-aerobically) and xylose. Additional strains containing both *xln43_SED1* and *xyn2* under different promoter combinations were then used to allow direct fermentation of beechwood xylan to ethanol in a CBP. The *SED1*_P_/*DIT1*_T_ and *TDH3*_P_/*DIT1*_T_ combinations significantly outperformed the benchmark *ENO1*_P/T_ under all of the tested cultivation conditions, as well as with regard to growth trials on non-native substrates (xylo-oligosaccharides/XOS and beechwood xylan) and fermentations of beechwood xylan to ethanol. Overall, *TDH3*_P_ was the best-performing promoter. This study demonstrates that heterologous metabolic pathways and CBP can be significantly enhanced by employing carefully selected promoters tailored to specific conditions.

**Key points:**

• *Promoters are unpredictable and must be tested under their intended conditions.*

• *TDH3*_*P*_, *SED1*_*P*_, *and DIT1*_*T*_ *were effective in enhancing heterologous xylanase activity.*

• *Optimized xylanolytic enzyme expression improved CBP of xylan to ethanol.*

**Supplementary Information:**

The online version contains supplementary material available at 10.1007/s00253-025-13563-6.

## Introduction

The baker’s yeast, *Saccharomyces cerevisiae*, has long been used in the production of fermented food and beverages, and has since emerged as a major microorganism to be used in industrial enzyme and biofuel production (Den Haan et al. 2021). While this yeast can produce first generation (1G) biofuels, such as bioethanol, from food crops (e.g. sugarcane, sugar beet, corn, etc.) with relative ease, the use of edible feedstocks for fuel production has raised controversy, sparking a “food vs fuel” debate due to concerns of additional pressure on the already-pressured food supply (Balan 2014; Lugani et al. 2020). This has led to the development of second generation (2G) biofuel production which makes use of non-edible lignocellulosic biomass (LCB) feedstocks to avoid encroaching on food resources (Olguin-Maciel et al. 2020). LCB is composed of cellulose (30–45%), hemicellulose (15–30%), and lignin (12–25%), with the exact composition varying depending on its source (Claes et al. 2020). The cellulose and hemicellulose portions of LCB contain sugars which can be fermented to bioethanol once these polymers have been hydrolysed. However, despite its high availability and renewability, the structural complexity of LCB prohibits a straightforward process to convert LCB to biofuel. Hemicellulose and lignin surround the cellulose fibres of LCB, making it recalcitrant and limiting the accessibility of hydrolysing enzymes. Thus, LCB requires pre-treatment to allow enzymes to hydrolyse the carbohydrate polymers to fermentable sugars (Mujtaba et al. 2023; Sharma et al. 2022).

After pre-treatment, enzyme production, saccharification, and fermentation steps must be performed to produce bioethanol (Kroukamp et al. 2018). With the ongoing improvements in biomass conversion technology, the combination of the aforementioned steps is envisioned to streamline the conversion of biomass to bioethanol in a process known as consolidated bioprocessing (CBP) where a single microorganism or microbial consortium is engineered to convert pre-treated biomass to bioproducts, without the need for exogenous enzymes (Davison et al. 2019; Jansen et al. 2017). *S. cerevisiae* is the preferred industrial ethanologen due to its robustness and tolerance to industrial stresses, high ethanol productivity, generally regarded as safe (GRAS) status, complete sequenced genome, abundance of omics data, and genetic amenability (Den Haan et al. 2021), but for its successful implementation in industrial CBP, it must be engineered to contain cellulolytic, xylanolytic, and xylose-metabolizing capabilities (Den Haan et al. 2013).

Cellulose remains the more attractive target for saccharification due to glucose being the most predominant sugar in LCB (Oh and Jin 2020). However, the utilization of hemicellulose for biofuel production is equally crucial. Xylan is the major component of hemicellulose and is made up of xylose monomers (among other sugars) with xylose second only to glucose as the most abundant sugar in lignocellulosic hydrolysates (Cunha et al. 2020; Oh and Jin 2020; Valenzuela-Ortega and French 2019; Xiao et al. 2019). Xylan consists of a β−1,4-linked xylose monomer backbone substituted with glucose, galactose, mannose, and arabinose side chains (Sakamoto et al. 2012; Van Dyk and Pletschke 2012). Removal of these side chains requires enzymes specific to the substrate, for example, α-arabinofuranosidase for arabinose side chains. Degradation of the xylan backbone requires two main xylanases, endo-β-xylanase, which hydrolyses the main xylan chain into xylo-oligosaccharides (XOS), and β-xylosidase to further cleave the XOS into xylose monomers (Cunha et al. 2020). Xylose must then enter cellular metabolism through a xylose-assimilating pathway. Two common pathways which have been introduced in *S. cerevisiae* are the oxidoreductive pathway (xylose reductase/xylitol dehydrogenase; XR/XDH) and the xylose isomerase pathway (XI), both of which enable the conversion of D-xylose to xylulose (Mert et al. 2017).

*S. cerevisiae* can be genetically modified to contain LCB-hydrolysing genes through methods such as CRISPR/Cas9, but a remaining drawback is its inherently low protein secretion capability (Jacob et al. 2022; Kroukamp et al. 2018). These low titres of secreted enzymes must be improved for CBP-enabled *S. cerevisiae* strains to be implemented on an industrial scale where large amounts of cellulases and/or xylanases are a necessity. Promoters are powerful genetic engineering tools, controlling the production of important enzymes throughout the metabolic network, and allowing for optimal microbial growth and/or desired product formation (Xu et al. 2019). As such, they have become an effective strategy for the manipulation and control of gene transcription with numerous reports on their enhancement of recombinant protein production across literature (Myburgh et al. 2020; Tang et al. 2020; Xiong et al. 2018; Yang et al. 2023). Endogenous promoters are broadly categorized as either inducible or constitutive. In the case of the former, transcription is initiated by certain environmental stimuli. Examples of such promoters include *GAL1/GAL7*_P_ and *GAL10*_P_, *CUP1*_P_, and *ADH2*_P_, induced by galactose, Cu^2+^, and the depletion of glucose, respectively (Peng et al. 2015; Tang et al. 2020). The induced spike in transcription associated with inducible promoters does not, however, imply that their expression is overall higher than their constitutive counterparts. In fact, Xiong et al. (2018) found that, in general, the constitutive promoters displayed higher strengths than the inducible promoters across varying conditions in *S. cerevisiae*. Similarly, it was reported by Li et al. (2019) that in strains engineered for lycopene production, constitutive promoters were more efficient in balancing lycopene synthesis and host metabolism than inducible promoters. Nevertheless, inducible promoters are useful in cases where the growth of the cell must be separated from product production, for example if the desired product(s), or its intermediaries, are toxic (Hubmann et al. 2014). It is more common for constitutive promoters to be used due to their relatively stable levels of gene expression across varying conditions (He et al. 2023). In metabolic engineering, widely used constitutive promoters include those of the glycolytic pathway such as *TDH3*_P_,* ENO2*_P_,* PGK1*_P_, *ADH1*_P_, and *PDC1*_P_, as well as *TEF1*_P_ and *CYC1*_P_ (He et al. 2023; Tang et al. 2020).

When designing heterologous expression systems, the choice of promoter is crucial, not only to achieve sufficient enhancement of recombinant protein production, but because promoter performance and regulation, even in so-called constitutive promoters, are notoriously subject to change under different environmental conditions (Den Haan et al. 2021). Even though a number of studies have compared the relative strength of promoters through expression of reporter genes such as yeast enhanced green fluorescent protein (*yEGFP*) or β-galactosidase (*lacZ*), in laboratory yeast strains on different substrates and under different conditions (Inokuma et al. 2014; Myburgh et al. 2020; Nambu-Nishida et al. 2018; Partow et al. 2010; Peng et al. 2015; Sun et al. 2012; Weinhandl et al. 2014; Xiong et al. 2018), variation in experimental setup was shown to affect comparability across these studies (Hubmann et al. 2014). Hence, instead of determining the overall “best” promoter, it may be more appropriate to investigate promoters under their specific intended conditions (Den Haan et al. 2021). The *TDH3* (glyceraldehyde-3-phosphate dehydrogenase; also referred to as *GDP*) promoter is widely regarded as the best native yeast promoter, with studies reporting it as one of the highest performing—if not the best—promoters in the enhancement of heterologous enzymes, both in the presence and absence of glucose (Myburgh et al. 2020; Nambu-Nishida et al. 2018; Partow et al. 2010; Xiong et al. 2018). A second promoter of interest is that of the *SED1* gene (encoding a stress-induced structural glycosylphosphatidylinositol cell wall protein) which has been shown to increase heterologous cellulase expression (Inokuma et al. 2014) as well as maintain moderate to high expression when grown on xylose-containing media, i.e. in the absence of glucose (Nambu-Nishida et al. 2018). Furthermore, terminators have served as additional tools to improve heterologous protein production of upstream coding genes. The terminator of the *DIT1* gene (encoding dityrsosine-deficient 1) has been reported as a strong terminator for the enhancement of heterologous protein production (Ito et al. 2016, 2013).

In a recent study by Kruger and Den Haan (2022), *Trichoderma reesei xyn2* and SED1-cell-tethered *Pyrenophora tritici-repentis xln43* were both successfully integrated and expressed under *ENO1*_P/T_ into *S. cerevisiae* S288C-MJM121 (engineered for xylose utilization via the xylose isomerase pathway). The constructed strains displayed heterologous enzyme production when cultivated on glucose, but little to no activity in a xylose medium. For *S. cerevisiae* strains to be viable for industrial applications such as 2G biofuels production, they must efficiently produce high yields of recombinant proteins on non-native substrates like xylose or under conditions with low glucose concentrations in low oxygen environments. Notably, the aforementioned *TDH3*_P_ and *SED1*_P_ have been reported to maintain high expression levels in the absence of glucose, and *DIT1*_T_ has been reported to further enhance the expression of upstream coding genes. Accordingly, this study investigated whether the promoters of the *S. cerevisiae TDH3* and *SED1* genes and terminator of the *DIT1* gene would yield superior heterologous *T. reesei xyn2* and *P. tritici-repentis xln43*_*SED1* expression in the absence of glucose or under fermentative conditions, using *ENO1*_P/T_ as a benchmark. Genetic engineering of these strains made use of a CRISPR/Cas9 methodology. Furthermore, we conducted growth trials on the engineered xylanase- and xylosidase-containing *S. cerevisiae* S288C-MJM121 strains on xylo-oligosaccharides (XOS) and beechwood xylan, and demonstrated the ability of these strains to directly ferment beechwood xylan to ethanol.

## Materials and methods

All chemicals and reagents used in this study were of laboratory grade and purchased from Sigma-Aldrich/Merck (St. Louis, MO, USA) unless otherwise stated.

### Plasmid isolation

Plasmids pRS42-G-ChX, pRSCG_11, pSED1p-DIT1t, pTDH3p-DIT1t, pRDH182, and pRDH177_SED1 (Table 1) were propagated in *Escherichia coli* DH5α (Thermo Fisher Scientific, Waltham, MA, USA). These strains were streaked out from 40% (v/v) glycerol stocks stored at − 80 °C onto Luria–Bertani (LB) agar (5 g/L yeast extract, 10 g/L tryptone, 10 g/L NaCl, and 20 g/L agar) supplemented with 100 µg/mL ampicillin and incubated at 37 °C overnight. To prepare for plasmid extraction, resulting colonies were inoculated in liquid LB supplemented with 100 µg/mL ampicillin and incubated at 37 °C on a rotary wheel overnight. Plasmid DNA was extracted using a Zyppy Plasmid Miniprep Kit (Zymo Research, Irvine, CA, USA) as directed by the manufacturer.

**Table 1:** Plasmids used in this study

Plasmid	Description	Reference
pRS42-G-ChX	gRNA scaffold plasmid targeting a yeast chromosome 10 intergenic sequence. Contains the G418 resistance marker	Jacob et al. (2022)
pRSCG_11	gRNA scaffold plasmid targeting a yeast chromosome 11 intergenic sequence. Contains the G418 resistance marker and a *cas9* gene cassette	Jacob et al. (2022)
pSED1p-DIT1t	pUC57s-SED1pDIT1t; plasmid with synthetic *SED1* promoter and *DIT1* terminator construct with *PacI*-*SgsI* recognition sites in between to allow cloning	GeneArt (Thermo Fisher Scientific)
pTDH3p-DIT1t	pUC57s-TDH3pDIT1t; plasmid with synthetic *TDH3* promoter and *DIT1* terminator construct with *PacI*-*SgsI* recognition sites in between to allow cloning	GeneArt (Thermo Fisher Scientific)
pRDH177_SED1	pMU1531-Ptrxld43; plasmid containing the *P. tritici-repentis xln43* gene with the *SED1* anchoring domain under control of the *ENO1* promoter and terminator	Kruger and Den Haan (2022)
pRDH182	pMU1531-nTrxyn2; plasmid containing the *T.* *reesei xyn2* gene under control of the *ENO1* promoter and terminator	Kruger and Den Haan (2022)
pSED1p-xln43_SED1-DIT1t	The *xln43*_*SED1* fusion gene (tethered xylosidase) cloned as a *PacI-SgsI* fragment into pSED1p-DIT1t	This study
pTDH3p-xln43_SED1-DIT1t	The *xln43*_*SED1* fusion gene (tethered xylosidase) cloned as a *PacI-SgsI* fragment into pTDH3p-DIT1t	This study
pSED1p-xyn2-DIT1t	The *xyn2* gene (xylanase) cloned as a *PacI-SgsI* fragment into pSED1p-DIT1t	This study
pTDH3p-xyn2-DIT1t	The *xyn2* gene (xylanase) cloned as a *PacI-SgsI* fragment into pTDH3p-DIT1t	This study

### Plasmid construction

The plasmids pSED1p-DIT1t and pTDH3p-DIT1t were ordered as synthetic constructs from GeneArt (Regensburg, Germany). The plasmids contain the promoter regions of the *S. cerevisiae SED1* or *TDH3* genes (SGD:S000002484 and SGD:S000003424) and the terminator sequence of the *DIT1* gene (SGD:S000002811) cloned onto pUC57 and separated by the recognition sequences for the restriction enzymes *Pac*I and *Sgs*I. To clone the relevant xylosidase and xylanase genes under the specified promoter (*SED1*_P_ or *TDH3*_P_) and terminator (*DIT1*_T_), vector plasmids (pSED1p-DIT1t, pTDH3p-DIT1t) and plasmids containing the genes of interest (pRDH177_SED1, pRDH182) were individually digested with restriction enzymes *Pac*I and *Sgs*I (Thermo Fisher Scientific). The digested DNA was resolved on a 1% (w/v) agarose gel. After confirmation of size, DNA was extracted and purified from the agarose gel as done by Thuring et al. (1975) with modifications, and further purified using a standard phenol, chloroform, isoamyl alcohol (PCI) clean up. In brief, DNA bands were cut from the agarose gel and forced through a syringe, suspended in an equal volume of phenol, and frozen at − 80 °C for 45 min. After centrifugation at 13,000 rpm for 15 min, the upper phase was collected and combined with an equal volume of PCI (made up in a 25:24:1 ratio) and then vortexed. After further centrifugation at 13,000 rpm for 2 min, the upper phase was collected and combined with an equal volume of chloroform:isoamyl alcohol (CI) (made up in a 24:1 ratio) and vortexed. Samples were subsequently centrifuged at 13,000 rpm for 1 min and the upper phase collected and precipitated with 1/10 volume 3 M sodium acetate and 2 volumes of 100% ethanol at − 20 °C overnight. DNA was then pelleted and washed with 70% ethanol. After drying, DNA was resuspended in deionized distilled water.

The isolated vector and insert DNA was ligated with T4 DNA ligase (Thermo Fisher Scientific) according to the manufacturer’s instructions, after which ligated DNA was dialyzed against deionized distilled water on a 0.025-µm MCE membrane (Merck Millipore, Burlington, MA, USA). Dialyzed DNA was combined with electro-competent *E. coli* DH5α cells in a chilled electroporation cuvette (Bio-Rad, Hercules, CA, USA) and transformed via electroporation using a Bio-Rad MicroPulser (25 µF capacitance, 2.5 kV, and 200 Ohm resistance). Cells were quickly resuspended in super optimal broth with catabolite repression (SOC) media (20 g/L tryptone, 5 g/L yeast extract, 10 mM NaCl, 2.5 mM KCl) and incubated at 37 °C with shaking for 1 h. Cells were plated on LB agar supplemented with 100 µg/mL ampicillin and incubated at 37 °C overnight. To confirm successful ligation and transformation, resulting colonies were propagated in LB broth supplemented with 100 µg/mL ampicillin at 37 °C overnight on a rotary shaker and the plasmid DNA extracted using a Zyppy Plasmid Miniprep Kit as per the manufacturer’s instructions. Plasmid DNA was digested with *Pac*I and *Sgs*I restriction enzymes and resolved on a 1% agarose gel to determine if both the plasmid vector and gene insert were present. This way, the *xln43_SED1* and *xyn2* genes were both cloned into the pSED1p-DIT1t and pTDH3p-DIT1t plasmids to create four new plasmids (pSED1p-xln43_SED1-DIT1t; pTDH3pxln43_SED1-DIT1t; pSED1p-xyn2-DIT1t; pTDH3p-xyn2-DIT1t) (Table 1).

### Strain construction

Constructed *xln43_SED1* gene cassettes were PCR-amplified using forward primers Ch10_SED1p-L or Ch10_TDH3p-L, and reverse primer Ch10_DIT1t-R (Table 2), generating homology repair templates specific to an intergenic sequence in chromosome 10 (Mikkelsen et al. 2012) to be used in the subsequent CRISPR/Cas9-based yeast transformation. All PCR reactions made use of Taq DNA Polymerase Master Mix RED (Ampliqon, Odense, Denmark) according to the manufacturer’s instructions. PCR reactions were set up with 31 cycles of 95 °C denaturation for 30 s, 60 °C annealing for 30 s, and 72 °C elongation for 2 min and 30 s. Similarly, constructed *xyn2* gene cassettes were amplified with forward primers Ch11_SED1p-L or Ch11_TDH3p-L, and reverse primer Ch11_DIT1t-R (Table 2) targeting chromosome 11 integration, with the only difference in the PCR reaction being the elongation time which was shortened to 2 min. PCR products were resolved on a 1% (w/v) agarose gel and purified using the PCI clean up. Purified DNA, as well as the plasmids containing CRISPR gRNA elements (pRS42-G-ChX, pRSCG_11; Table 1), was quantified on a NanoDrop 2000 spectrophotometer (Thermo Fisher Scientific) and dialyzed against deionized distilled water on a 0.025-µm MCE membrane.

**Table 2:** Primers used in this study

Primer	Sequence (5′−3′)	Function
Ch10_SED1p-L	GCAGTTATCTCTGTGTCCAGATCCCTTTGAAGTAAAGTTTATTGGATATAGAAAATTAACGTAAGGCAGTATC	To amplify genes under *SED1*_P_ and allow its targeting to the Ch10 integration site (used with Ch10_DIT1t-R)
Ch10_TDH3p-L	GCAGTTATCTCTGTGTCCAGATCCCTTTGAAGTAAAGTTTTTCAGTTCGAGTTTATCATTATCAATACTGCC	To amplify genes under *TDH3*_P_ and allow its targeting to the Ch10 integration site (used with Ch10_DIT1t-R)
Ch10_DIT1t-R	CTACAGTAATTGTGCGGTGCAGGGAGGCAATGTTTAGTGCTTACTCCGCAACGCTTTTCTG	To amplify genes under *SED1*_P_/*TDH3*_P_ and *DIT1*_T_ and allow its targeting to the Ch10 integration site
Ch11_SED1p-L	TGTAAAACAGGTATTGGCTGCTTCATAGTACACCCAATTGATTGGATATAGAAAATTAACGTAAGGCAGTATC	To amplify genes under *SED1*_P_ and allow its targeting to the Ch11 integration site (Used with Ch11_DIT1t-R)
Ch11_TDH3p-L	GCAACTCTGAAATGTCAAAACGGTCGTGTATAAATAAATGTTACTCCGCAACGCTTTTCTG	To amplify genes under *TDH3*_P_ and allow its targeting to the Ch11 integration site (Used with Ch11_DIT1t-R)
Ch11_DIT1t-R	TGTAAAACAGGTATTGGCTGCTTCATAGTACACCCAATTGTTCAGTTCGAGTTTATCATTATCAATACTGCC	To amplify genes under *SED1*_P_/*TDH3*_P_ and *DIT1*_T_ and allow its targeting to the Ch11 integration site

The *S. cerevisiae* S288C-MJM121 strain (M) used (Table 3) was a xylose-utilizing strain described by Mert et al. (2017). This strain was streaked out from a 15% (v/v) glycerol stock onto YPD agar (10 g/L yeast extract, 20 g/L peptone, 20 g/L glucose, and 20 g/L agar) and grown at 30 °C for 48 h. A single colony was inoculated into 20 mL YPD broth and incubated in an aerated conical flask at 30 °C at 180 rpm on an orbital shaker overnight. Yeast cells were harvested from the overnight culture and washed with deionized distilled water. To make the yeast cells electro-competent, washed cells were suspended in LiOAc/TE solution (100 mM LiOAc, 10 mM Tris–HCl pH 8.0, and 1 mM EDTA) and incubated at 30 °C at 180 rpm for 45 min. Subsequently, 20 µL 1 M dithiothreitol (DTT) was added to the mixture, which was incubated at 30 °C at 180 rpm for a further 15 min. The mixture was centrifuged, and the cells washed with distilled deionized water, then with electroporation buffer (1 M sorbitol and 20 mM HEPES). The yeast cells were transformed using approximately 10 µg of the respective DNA repair template and 1 µg CRISPR-based integration plasmid DNA with an electric pulse of 25 µF capacitance, 1.4 kV, and 200 Ohms. In this CRISPR/Cas9-based transformation, strains had the *xln43*_*SED1* gene cassettes targeted to chromosome 10, and the *xyn2* gene cassettes targeted to chromosome 11. Electroporated cells were resuspended in YPD broth supplemented with 1 M sorbitol. After both a 3-h and overnight incubation, the transformation mix was plated on YPD agar supplemented with 200 µg/mL Geneticin (G418) and incubated at 30 °C for 48 h.

**Table 3:** Yeast strains used in this study

Yeast strain	Abbreviation	Description	Reference
S288C-MJM121	M	Recombinant xylose-utilizing strain	Mert et al. (2017)
S288C-MJM121 + pcasNAT	M-Cas	Recombinant xylose-utilizing strain with the plasmid containing the *cas9* cassette	Kruger and Den Haan (2022)
YI13 + *SED1*_P_-*xyn2*-*DIT1*_T_	Y_S-xyn	YI13 strain containing *T. reesei xyn2* under control of *SED1*_P_ and *DIT1*_T_ used as positive control	This laboratory
*ENO1*_P_-*xln43*_*SED1*-*ENO1*_T_ Ch10	E-xln	Xylose-utilizing strain with Ch10 CRISPR-integrated *P. tritici-repentis xln43*_*SED1* under control of *ENO1*_P/T_	Kruger and Den Haan (2022)
*ENO1*_P_-*xyn2*-*ENO1*_T_ Ch11	E-xyn	Xylose-utilizing strain with Ch11 CRISPR-integrated *T. reesei xyn2* under control of *ENO1*_P/T_	Kruger and Den Haan (2022)
*ENO1*_P_-*xln43*_*SED1*-*ENO1*_T_ Ch10 + *ENO1*_P_-*xyn2*-*ENO1*_T_ Ch11	E-xln_E-xyn	Xylose-utilizing strain with Ch10 CRISPR-integrated *P. tritici-repentis xln43*_*SED1* and Ch11 CRISPR-integrated *T. reesei xyn2*, both under control of *ENO1*_P/T_	Kruger and Den Haan (2022)
*SED1*_P_-*xln43*_*SED1*-*DIT1*_T_ Ch10	S-xln	Xylose-utilizing strain with Ch10 CRISPR-integrated *P. tritici-repentis xln43*_*SED1* under control of *SED1*_P_ and *DIT1*_T_	This study
*TDH3*_P_-*xln43*_*SED1*-*DIT1*_T_ Ch10	T-xln	Xylose-utilizing strain with Ch10 CRISPR-integrated *P. tritici-repentis xln43*_*SED1* under control of *TDH3*_P_ and *DIT1*_T_	This study
*SED1*_P_-*xyn2*-*DIT1*_T_ Ch11	S-xyn	Xylose-utilizing strain with Ch11 CRISPR-integrated *T. reesei xyn2* under control of *SED1*_P_ and *DIT1*_T_	This study
*TDH3*_P_-*xyn2*-*DIT1*_T_ Ch11	T-xyn	Xylose-utilizing strain with Ch11 CRISPR-integrated *T. reesei xyn2* under control of *TDH3*_P_ and *DIT1*_T_	This study
*TDH3*_P_-*xln43*_*SED1*-*DIT1*_T_ Ch10 + *SED1*_P_-*xyn2*-*DIT1*_T_ Ch11	T-xln_S-xyn	Xylose-utilizing strain with Ch10 CRISPR-integrated *P. tritici-repentis xln43*_*SED1* under control of *TDH3*_P_ and *DIT1*_T_ and Ch11 CRISPR-integrated *T. reesei xyn2* under control of *SED1*_P_ and *DIT1*_T_	This study
*TDH3*_P_-*xln43*_SED1-*DIT1*_T_ Ch10 + *TDH3*_P_-*xyn2*-*DIT1*_T_ Ch11	T-xln_T-xyn	Xylose-utilizing strain with Ch10 CRISPR-integrated *P. tritici-repentis xln43*_*SED1* and Ch11 CRISPR-integrated *T. reesei xyn2*, both under control of *TDH3*_P_ and *DIT1*_T_	This study

### Confirmation of successful transformation

Putative positive *xln43_SED1* transformants were inoculated in 1.5 mL YPD broth in a 96-deep-well plate and sealed with a breathable AeraSeal™ film and incubated at 30 °C at 180 rpm for 48–72 h. These small-scale cultures were then screened for xylosidase activity on the substrate *p*-nitrophenyl-β-D-xylopyranoside (pNP-X). Cell culture was combined with 250 mM pNP-X and 50 mM NaOAc buffer in a 5:1:44 ratio to a 100 µL volume. This mixture was incubated at 50 °C for 30 min and the reaction was stopped by the addition of 100 µL 1 M sodium carbonate. Confirmation of positive xln43_*SED1* transformants was indicated by a yellow colour formation.

The confirmation of *xyn2* transformants was done by spot plating putative transformant colonies onto SC^−HIS^ agar (1.7 g/L yeast nitrogen base, 20 g/L glucose, 5 g/L ammonium sulfate, 1.5 g/L amino acids without histidine, and 20 g/L agar) supplemented with 1 g/L azurine cross-linked (AZCL)-xylan (Megazyme, Bray, Ireland) and incubating at 30 °C overnight. Positive *xyn2* transformants were indicated by the presence of a dark blue halo surrounding the colony (Cedras et al. 2020). A previously constructed *S. cerevisiae* YI13 strain containing *T. reesei xyn2* was used as a positive control. Notable strains constructed in this study were submitted to the publicly accessible Biobanks South Africa Yeast Culture Collection at the Department of Microbiology and Biochemistry, University of the Free State. Strain collection numbers are detailed in the supplementary material (Supplementary Table S1).

### Enzyme activity assays

Overnight YPD pre-cultures of the engineered *xln43_SED1* and *xyn2* expressing strains were used to inoculate, in triplicate, 10 mL YPD in conical flasks or 20 mL YPD in rubber-stoppered bottles containing a glass bead to aid in culture mixing. Rubber-stoppered bottles were also pierced with sterilized 0.8 × 25 mm hypodermic needles stopped with cotton wool to allow for CO_2_ release while maintaining a micro-aerobic environment. Similarly, overnight YPX (10 g/L yeast extract, 20 g/L peptone, 20 g/L xylose) cultures of the engineered strains were made, which underwent an additional two sub-cultures before inoculating, in triplicate, into conical flasks with 10 mL YPX media. The sub-culturing was deemed necessary to allow sufficient growth and adaptation to xylose, which is not the preferred carbon source of the yeast strain used in this study. The YPD flasks, YPD rubber-stoppered bottles, and YPX flasks were incubated at 30 °C at 180 rpm for 72 h to determine xylosidase and xylanase enzymatic activity on glucose, under fermentative conditions, and on xylose, respectively.

The enzyme activity assays were all measured in triplicate. Measurement of cell-tethered xylosidase activity of the total cell culture was done using pNP-X substrate as outlined above in the preliminary screening with additional steps. After the addition of 1 M sodium carbonate, the mixture was centrifuged at 3000 rpm for 2 min, and 100 µL of the supernatant was transferred to a 96-well microtitre plate, and the absorbance was measured at 400 nm on a FLUOstar Omega Microplate Reader (BMG LABTECH, Ortenberg, Germany). The results were compared to a generated *p*-nitrophenyl (pNP) standard curve set between 0.075 mM and 1.25 mM to determine the amount of pNP released as a measure of xylosidase activity.

Evaluation of secreted xylanase activity was done using the dinitrosalicyclic acid (DNS) method as described by Bailey et al. (1992). Cell cultures were centrifuged at 3000 rpm for 2 min, and the supernatant was collected. The supernatant was combined with 10 g/L beechwood xylan (Megazyme) substrate (which had been made up to its final volume with 50 mM NaOAc buffer, boiled, and left to stir overnight) and incubated at 50 °C for 30 min. After the subsequent addition of DNS, the mixture was further incubated at 90 °C for 5 min, then 4 °C for 1 min. The final volume was 140 µL with a supernatant: substrate: DNS ratio of 1: 5: 8. Background sugar was determined by adding DNS to the supernatant before the addition of the xylan substrate to stop interaction between the xylanase enzyme and the beechwood xylan substrate, followed by the incubation step at 90 °C for 5 min, then 4 °C for 1 min. A 100 µL volume of each mixture was transferred to a 96-well microtitre plate, and the absorbance was measured at 540 nm spectrophotometrically on a FLUOstar Omega Microplate Reader. A standard curve was generated using xylose concentrations ranging from 0.5 g/L to 15 g/L. With high xylanase activity being observed in the cultures grown on YPD, the 50 °C incubation time was decreased to 5 min and supernatant diluted as necessary to stay in the range of the standard curve.

The OD_600_ of the grown cultures was measured to determine the DCW (g/L) of each culture (Jacob et al. 2022) to calculate enzyme activity as U/gDCW. The units of enzyme activity were expressed as U/L where one unit was defined as the amount of enzyme required to produce 1 µmol reducing sugar or equivalent per min (µmol/min). The calculated enzyme activity was then divided by the DCW (g/L) to get units of U/gDCW [(µmol/min)/L per gDCW].

### Co-producing xylosidase and xylanase strain construction and enzymatic assays

Based on the results of the enzymatic assays, S288C-MJM121-based strains were constructed to contain both *P. tritici-repentis xln43*_*SED1* targeted to chromosome 10, and *T. reesei xyn2* to chromosome 11. These strains made use of what was determined to be the strongest tested promoter: *TDH3*_P_ for both genes. Additionally, a second strain was constructed which made use of *TDH3*_P_ (for the *xln43_SED1*) and *SED1*_P_ (for the *xyn2*) to avoid potential overuse of *TDH3*_P_-related transcription machinery which may slow down the transcription process. These strains were constructed using the exact same methodology as stated above, except the host strain had been changed to a *S. cerevisiae* S288C-MJM121 strain (M-Cas) (Table 3) which was based on the same xylose-utilizing strain (Mert et al. 2017) and had been previously transformed with the pCas9-Nat plasmid to allow for CRISPR/Cas9-based engineering (Kruger and Den Haan 2022). M-Cas was streaked from a 15% (v/v) glycerol stock onto YPD agar supplemented with 100 µg/mL Nourseothricin (CloNAT) (Jena Bioscience, Jena, Germany). A single colony was then inoculated into 20 mL YPD broth supplemented with 100 μg/mL CloNAT and incubated in an aerated conical flask at 30 °C at 180 rpm on an orbital shaker overnight before electrotransformation (using the same methodology described above) with the constructed pTDH3p-xln43_SED1-DIT1_t_ targeted to chromosome 10. The transformation mix was then plated on YPD agar supplemented with 100 μg/mL CloNAT and 200 μg/mL G418. After successfully screening for positive transformants using the previously described pNP-X assay, the newly constructed strain containing *xln43_SED1* under *TDH3*_P_/*DIT1*_T_ underwent 3–5 rounds of sub-culturing on YPD agar supplemented with 100 µg/mL CloNAT with 48 h in between each sub-culture to remove the remaining CRISPR-based integration plasmid DNA. The same CRISPR/Cas9-based methodology was used to insert the relevant *xyn2* construct (pSED1p-xyn2-DIT1t or pTDH3p-xyn2-DIT1t; Table 1) into the yeast genome. Confirmation of successful transformation was done as previously stated using SC-based agar plates containing AZCL-xylan. This resulted in an additional two strains: T-xln_S-xyn and T-xln_T-xyn (Table 3). Strains containing both *xln43*_*SED1* and *xyn2* were then subjected to xylosidase and xylanase assays after cultivation on YPD, YPX, and under fermentative conditions. A strain containing the same genes, both under *ENO1*_P/T_, described in a previous report (Kruger and Den Haan 2022), was used as a benchmark strain against the newly constructed strains.

### Growth trials

For growth trials on glucose- and xylose-containing media, strains were pre-cultured in their respective media (YPD or YPX), containing 100 µg/mL ampicillin and 100 µg/mL streptomycin (Thermo Fisher Scientific) to suppress bacterial growth, for 24 h at 30 °C and 180 rpm. The pre-cultures were used to inoculate flasks containing 10 mL YPD or YPX supplemented with 100 µg/mL ampicillin and 100 µg/mL streptomycin to an OD_600_ of 0.05. OD_600_ measurements were taken at regular intervals on a FLUOstar Omega Microplate Reader, and the resulting values converted to DCW (g/L).

To evaluate the growth on XOS and xylan substrates, strains producing both xylosidase and xylanase enzymes were pre-cultured in YPX media supplemented with 100 µg/mL ampicillin and 100 µg/mL streptomycin for 24 h. Following this, the strains were sub-cultured twice in fresh YPX media supplemented with 100 µg/mL ampicillin and 100 µg/mL streptomycin (Thermo Fisher Scientific) to an OD_600_ of 0.05 with 24 h in between each sub-culture.

Measurement of growth on XOS and xylan was done using the same methodology as described by Kruger and Den Haan (2022). To measure growth on XOS, pre-cultured strains were inoculated into conical flasks containing 10 mL SC^−URA^ media without glucose supplemented with 50 g/L XOS (Carl Roth, Karlsruhe, Germany) to an OD_600_ of 0.05 and incubated at 30 °C shaking at 180 rpm. OD_600_ measurements were taken at regular intervals (every 24 h) over 16 days (384 h) on a FLUOstar Omega Microplate Reader, and the resulting values converted to DCW (g/L).

For growth on xylan, the same pre-cultured strains were inoculated into flasks containing 10 mL SC^−URA^ media without glucose supplemented with 50 g/L beechwood xylan (Megazyme) to an OD_600_ of 0.05 and incubated at 30 °C shaking at 180 rpm. Samples were taken at regular intervals (every 24 h), diluted as necessary and counted under a compound light microscope using a Neubauer-improved haemocytometer counting chamber (Paul Marienfeld GmbH & Co. KG, Lauda-Königshofen, Germany), as described by Thomson et al. (2015), over 24 days (576 h). Cell counts were then converted to cells/mL before being converted to DCW (g/L).

The remaining culture of both the XOS and xylan growth trials was subjected to a phenol–sulfuric acid assay to determine the remaining XOS and xylan in the flask culture. Samples were centrifuged at 13,000 rpm for 10 min, and 200 µL of cell-free supernatant was combined with 200 µL 5% (w/v) phenol solution in a glass test tube in triplicate. Samples were vortexed gently, followed by the addition of 800 µL concentrated sulfuric acid and additional vortexing. 200 µL sample was transferred to a 96-well microtitre plate, and the absorbance was measured at 480 nm on a FLUOstar Omega Microplate Reader. XOS and xylan standard curves were generated using concentrations ranging from 0.5 to 8 g/L. The remaining sugar was calculated in g/L.

### Xylan fermentation

The best-performing strain (T-xln_T-xyn), based on enzymatic assay results, was evaluated for the production of ethanol from beechwood xylan (Megazyme) in a CBP along with E-xln_E-xyn which served as a benchmark strain. YPX pre-cultures of these co-producing strains were grown for 24 h and were used to inoculate 50 mL YPX media supplemented with 100 µg/mL ampicillin and 100 µg/mL streptomycin to OD_600_ 0.025. These cultures were incubated at 30 °C with shaking at 180 rpm for 96 h. Rubber-stoppered bottles containing 10 mL double-strength YP media (20 g/L yeast extract, 40 g/L peptone) supplemented with 80 g/L beechwood xylan (Megazyme) were inoculated in biological triplicate with 10 mL of the YPX flask culture (to achieve a final concentration of 40 g/L beechwood xylan). A sterilized glass bead was added to each bottle to improve the mixing of the fermentation broth. The rubber-stoppered bottles were pierced with sterilized 0.8 × 25 mm hypodermic needles plugged with cotton wool to allow for CO_2_ release while maintaining a micro-aerobic environment. The fermentation bottles were incubated at 30 °C with shaking at 180 rpm for 144 h, with 2 mL samples being taken at 0, 72, 120, and 144 h. These samples were centrifuged at 13,000 rpm for 10 min, and the cell-free supernatant was stored at − 20 °C until further analysis.

1 mL of the thawed supernatant was transferred to a clean Eppendorf tube and acidified with 50 µL 10% (v/v) sulfuric acid and vortexed briefly to ensure mixing. Samples were filtered through a 0.22-µm filter into 2 mL high-performance liquid chromatography (HPLC) vials. Ethanol, xylose, acetic acid, and glycerol concentrations were determined in each sample by an HPLC equipped with a Bio-Rad guard (part # 125–0129) 7.8 × 300 mm column and refractive index (RI) detector at a temperature of 65 °C with 5 mM sulfuric acid as the mobile phase at a flow rate of 0.7 mL/min.

### Statistical analysis

All values obtained were presented as the mean of biological triplicates with their standard deviations. Significant differences between enzyme activities, growth data, and/or metabolite concentrations attained were investigated using two-tailed unpaired T-tests assuming unequal variance. A *p*-value < 0.05 was deemed significant. Calculations were done using Microsoft Excel.

## Results

### Strain construction

In this study, we evaluated the ability of various promoters and a strong terminator to drive the expression of genes encoding xylanolytic enzymes under conditions that better mimic industrial processes, specifically low glucose and low oxygen concentrations. Additionally, we investigated whether enhanced xylanolytic enzyme production could improve growth on polymeric substrates and increase the efficiency of xylan conversion to ethanol. *S. cerevisiae* S288C-MJM121, previously engineered for xylose utilization (Mert et al. 2017), was transformed either with *P. tritici-repentis xln43*_*SED1* or *T. reesei xyn2* under the control of either the *SED1* or *TDH3* promoters and *DIT1* terminator. Two more xylose-utilizing S288C-MJM121 strains, engineered to contain both *xln43_SED1* and *xyn2*, were also constructed: both strains were transformed with *xln43*_*SED1* under *TDH3*_P_/*DIT1*_T_, but one strain was additionally transformed with *xyn2* under *SED1*_P_/*DIT1*_T_ and the other with *xyn2* under *TDH3*_P_/*DIT1*_T_. All *xln43*_*SED1* and *xyn2* cassettes were targeted to intergenic loci on chromosomes 10 and 11, respectively, via a CRISPR/Cas9-based strategy. This study made use of a GH43 xylosidase tethered to the cell wall by the SED1 anchoring domain, which previously displayed higher enzyme activity compared to a secreted xylosidase (Kruger and Den Haan 2022). The GH11 xylanase-encoding gene used produced a xylanase efficiently secreted into the growth media.

**Fig. 1:**
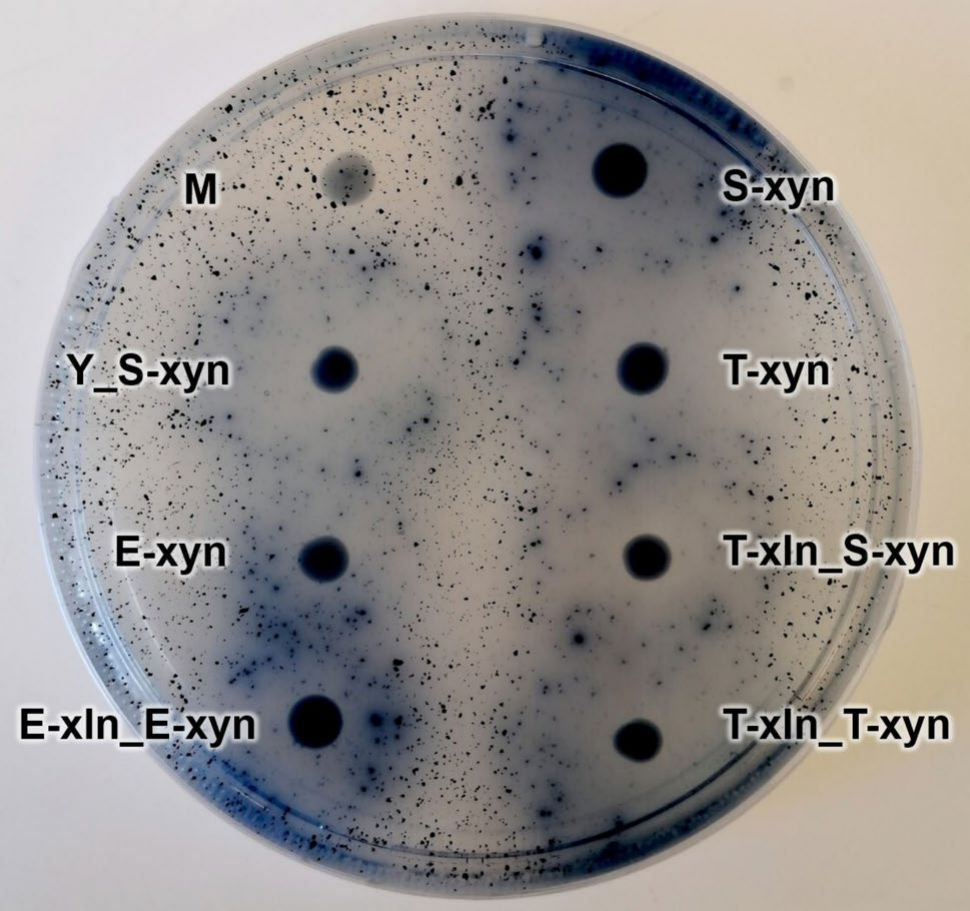
Engineered *S. cerevisiae* strains containing *T. reesei xyn2* spot-plated on a 0.1% AZCL-xylan agar plate with xylanase activity indicated by a surrounding blue halo

The *xln43_SED1* cassettes were successfully transformed into the xylose-utilizing *S. cerevisiae* strain (M) through CRISPR/Cas9. This was confirmed through a small-scale preliminary assay using pNP-X substrate. Xylosidase cleaves the pNP-X substrate, liberating the pNP which is yellow in colour; hence, positive *xln43*_*SED1* transformants displayed a yellow colour during screening (result not shown). Confirmation of successful *xyn2* transformation into *S. cerevisiae* S288C-MJM121 was done using an AZCL-xylan plate assay. Putative *xyn2* transformants were spot-plated on AZCL-xylan plates to detect xylanase activity. Upon cleavage of AZCL-xylan to XOS and xylose by xylanase, dark blue dye molecules are released from the polysaccharide. Therefore, strains positive for xylanase activity are indicated by a blue halo surrounding the spot-plated colony (Fig. 1).

The presence of xylosidase and xylanase activity in the respective engineered strains produced in this study indicated the ability of the chosen promoters (*SED1*_P_ and *TDH3*_P_) and terminator (*DIT1*_T_) to successfully express the *P. tritici-repentis xln43*_*SED1* and *T. reesei xyn2* genes when integrated at chromosomes 10 and 11, respectively, through a CRISPR/Cas9 strategy. Subsequently, enzyme activity was quantified through their respective assays.

### Enzymatic assays

The employment of strong promoters for the improved expression of heterologous genes in yeast has become a common practice (Partow et al. 2010). Although constitutive promoters were initially thought to perform independently of environmental conditions and maintain relatively constant gene expression, it has been determined that numerous factors, including experimental setup, cloning strategies, reporter proteins, and genetic background, influence the transcriptional activation in constitutive promoters (Myburgh et al. 2023). Therefore, the evaluation of promoter performance with a specific reporter protein of interest and under the environmental conditions for which it is intended has become warranted. The activity of *SED1*_P_/*DIT1*_T_ and *TDH3*_P_/*DIT1*_T_ under the chosen conditions was assessed by quantifying the activity of xylosidase and xylanase, whose genes were cloned under their control. *S. cerevisiae* strains containing the same *xln43*_*SED1* and *xyn2* genes under *ENO1*_P/T_, individually and in combination (Kruger and Den Haan 2022), were used as benchmark strains. While yeast cultivation on glucose media, both aerobically and under fermentative conditions, is relatively simple, xylose is a non-native substrate for *S. cerevisiae*. However, the *S. cerevisiae* S288C-MJM121 host strain used in this study was previously engineered to contain a xylose isomerase pathway, enabling the assimilation of xylose through the pentose phosphate pathway (Cunha et al. 2019; Lane et al. 2018). Thus, the engineered yeast strains in this study could grow sufficiently in xylose media, albeit requiring pre-culturing to adapt to this carbon source. Xylosidase and xylanase activities were quantified through pNP-X- and DNS-based assays, respectively, after shake flask cultivations, and in the case of fermentative conditions, cultivation in rubber-stoppered bottles.

#### Single-gene strain enzymatic assays

**Fig. 2:**
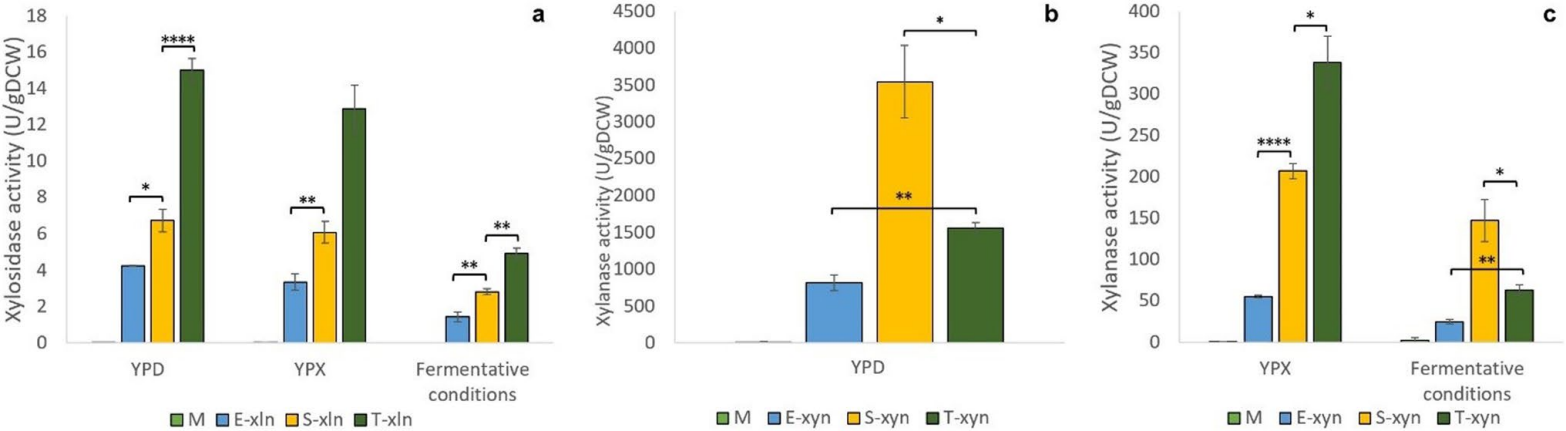
Heterologous enzyme activity (U/gDCW) of *S. cerevisiae* S288C-MJM121 strains engineered to contain **a** *P. tritici-repentis SED1*-cell-tethered *xln43* and **b**, **c** *T. reesei xyn2* under different promoters and terminators, after 72 h of cultivation in YPD and YPX and under fermentative conditions. Data represents the means of triplicate cultures, with error bars indicating standard deviation. Asterisks are used to indicate *p*-values calculated using the unpaired t-test assuming unequal variance: *p < 0.05; **p < 0.01; ***p < 0.001; and ****p < 0.0001

The untransformed M strain showed no enzyme activity, indicating no natural xylosidase or xylanase activity in this strain (Fig. 2). After assaying yeast strains containing a single gene (*xln43*_*SED1* or *xyn2*), we observed that engineered strains produced the highest activity when cultivated aerobically in YPD media, followed by YPX media, with their lowest activity detected when cultivated under fermentative conditions. In the case of xylosidase activity, both *SED1*_P_/*DIT1*_T_ and *TDH3*_P_/*DIT1*_T_ significantly outperformed the benchmark *ENO1*_P/T_ strain, with *TDH3*_P_ furthermore significantly outperforming *SED1*_P_ (except when cultivated on YPX media) (Fig. 2a). *SED1*_P_/*DIT1*_T_ achieved levels of xylosidase activity that were between 1.5- to 2-fold higher than the benchmark *ENO1*_P/T_ under the three chosen growth conditions. Even more impressively, *TDH3*_P_/*DIT1*_T_-based expression yielded 3.75- to 4-fold more xylosidase activity than *ENO1*_P/T_-based expression in aerobic cultivations on glucose and xylose media, as well as under fermentative conditions. *TDH3*_P_ outperformed *SED1*_P_ by factors of 2–2.5 across the three cultivation conditions.

Results of the evaluated xylanase activity showed that, once again, *SED1*_P_/*DIT1*_T_ and *TDH3*_P_/*DIT1*_T_ both significantly outperformed *ENO1*_P/T_-based expression (Fig. 2b, c). However, in contrast to the xylosidase activity result, *SED1*_P_ significantly outperformed *TDH3*_P_ when cultivated on glucose media and under fermentative conditions. Nevertheless, *TDH3*_P_/*DIT1*_T_ remained the best promoter/terminator combination when strains were cultivated on YPX. For strains cultivated on glucose (aerobically and micro-aerobically) there was an approximate 2-fold difference in xylanase activity between *ENO1*_P/T_ and *TDH3*_P_/*DIT1*_T_, and *TDH3*_P_/*DIT1*_T_ and *SED1*_P_/*DIT1*_T_ strains. After cultivation in xylose media, there was a difference in xylanase activity of 3.8-fold between *ENO1*_P/T_ and *SED1*_P_/*DIT1*_T_, and 1.5-fold between the *SED1*_P_/*DIT1*_T_ and *TDH3*_P_/*DIT1*_T_ strains.

#### Co-producing xylosidase and xylanase strain enzymatic assays

**Fig. 3:**
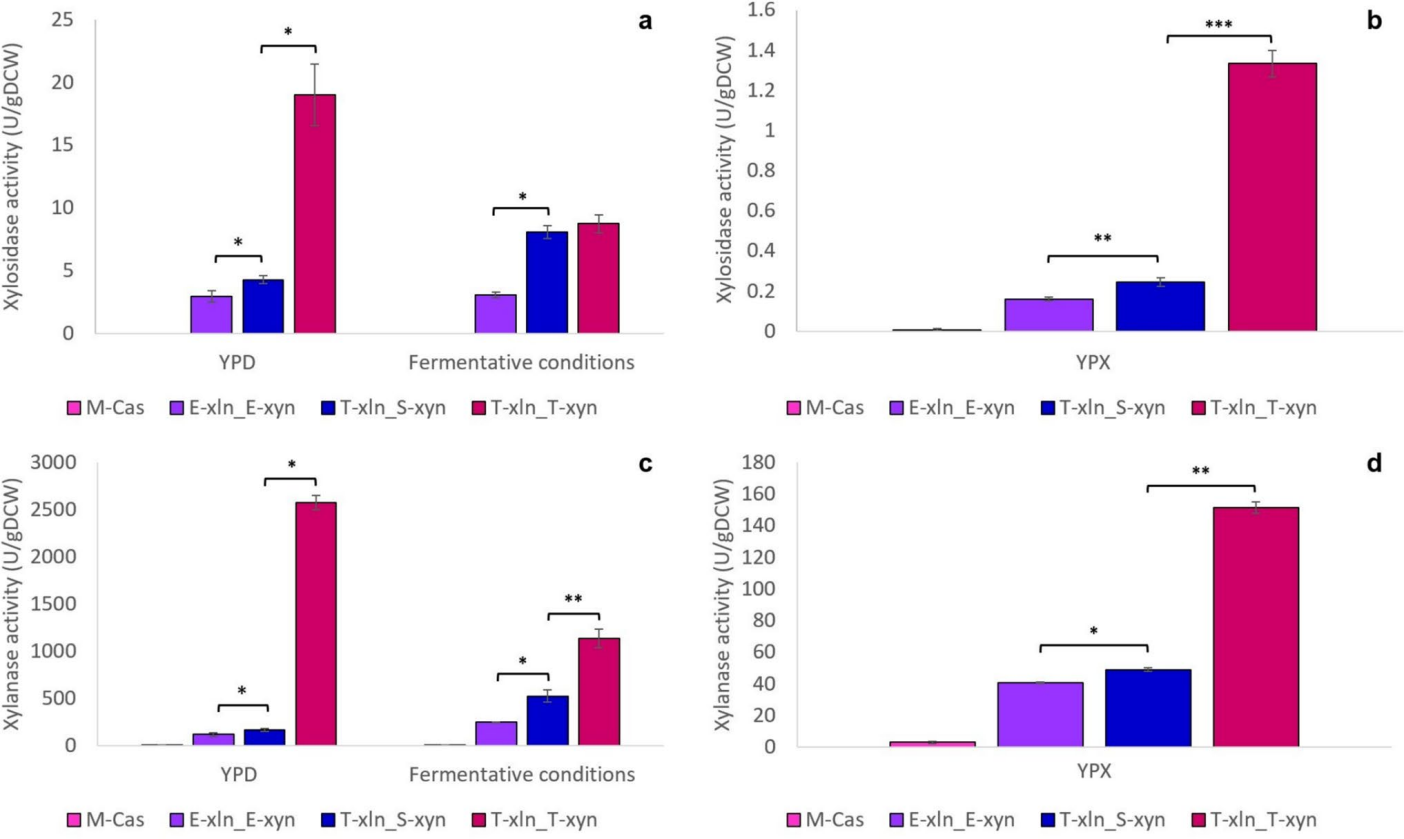
Heterologous enzyme activity (U/gDCW) of *S. cerevisiae* S288C-MJM121 strains engineered to contain *P.* *tritici-repentis* SED1-cell-tethered *xln43* and *T. reesei xyn2* under different promoter and terminator combinations after 72 h of cultivation. β-xylosidase activity of strains cultivated on **a** YPD and under fermentative conditions, and **b** YPX. Endo-β-xylanase activity of strains cultivated on **c** YPD and under fermentative conditions, and **d** YPX. Data represents the means of triplicate cultures, with error bars indicating standard deviation. Asterisks are used to indicate *p*-values calculated using the unpaired t-test assuming unequal variance: *p < 0.05; **p < 0.01; ***p < 0.001; and ****p < 0.0001

As with strains containing only one heterologous gene, strains containing both genes under the *SED1* and/or *TDH3* promoters and *DIT1* terminator significantly outperformed the benchmark strain containing both genes under *ENO1*_P/T_ (Fig. 3). When analysing xylosidase activity in strains containing both genes, significant differences in enzyme activity were observed among strains with different promoter combinations (Fig. 3a, b). However, when cultivated on xylose media, all strains exhibited notably lower enzyme activity (< 1.4 U/gDCW) compared to strains containing only *xln43_SED1* (Figs. 2 and 3).

T-xln_S-xyn and T-xln_T-xyn both produced significantly higher xylosidase activity than E-xln_E-xyn by factors of 2 and 9.5 on YPD, 1.5 and 8.4 on YPX, respectively, and both by a factor of 2.7 under fermentative conditions (Fig. 3a, b). Interestingly, there was a significant difference in xylosidase activity between the T-xln_S-xyn and T-xln_T-xyn strains, except when cultivated under fermentative conditions. This was surprising as the *xln43*_*SED1* gene was cloned under the same promoter and terminator (*TDH3*_P_/*DIT1*_T_) in both strains. Yet, T-xln_T-xyn strains displayed 4.8- and 5.6-fold higher xylosidase activity than T-xln_S-xyn when cultivated aerobically on glucose and xylose, respectively.

Results of xylanase activity assays showed the same trend as the xylosidase activities where strains displayed notably lower levels of enzyme activity when cultivated on xylose media, as well as both T-xln_S-xyn and T-xln_T-xyn significantly outperforming E-xln_E-xyn (Fig. 3c, d). The T-xln_T-xyn strain displayed the highest level of xylanase activity when cultivated on glucose, xylose, and under fermentative conditions, significantly outperforming even T-xln_S-xyn by factors of 15.6, 3.1, and 2.2, respectively. Despite strains containing only *xyn2* under *SED1*_P_ having higher xylanase activity than those under *TDH3*_P_, it appeared that, when expressing both *xyn2* and *xln43*_*SED1*, the strain which made use of *TDH3*_P_ for both genes was superior, significantly outperforming T-xln_S-xyn.

### Growth trials

Strains containing both heterologous xylanolytic genes underwent growth trials on monomeric sugars. Strains were cultivated in YP media containing 20 g/L glucose or 20 g/L xylose (Fig. S1). No significant differences in growth were observed when the strains were cultivated on glucose as a carbon source. A slight difference was observed when strains were cultivated on YPX, as the T-xln_S-xyn and T-xln_T-xyn strains reached stationary phase earlier (~ 20 h before) than the E-xln_E-xyn and M-Cas strains, after which biomass reached similar levels.

**Fig. 4:**
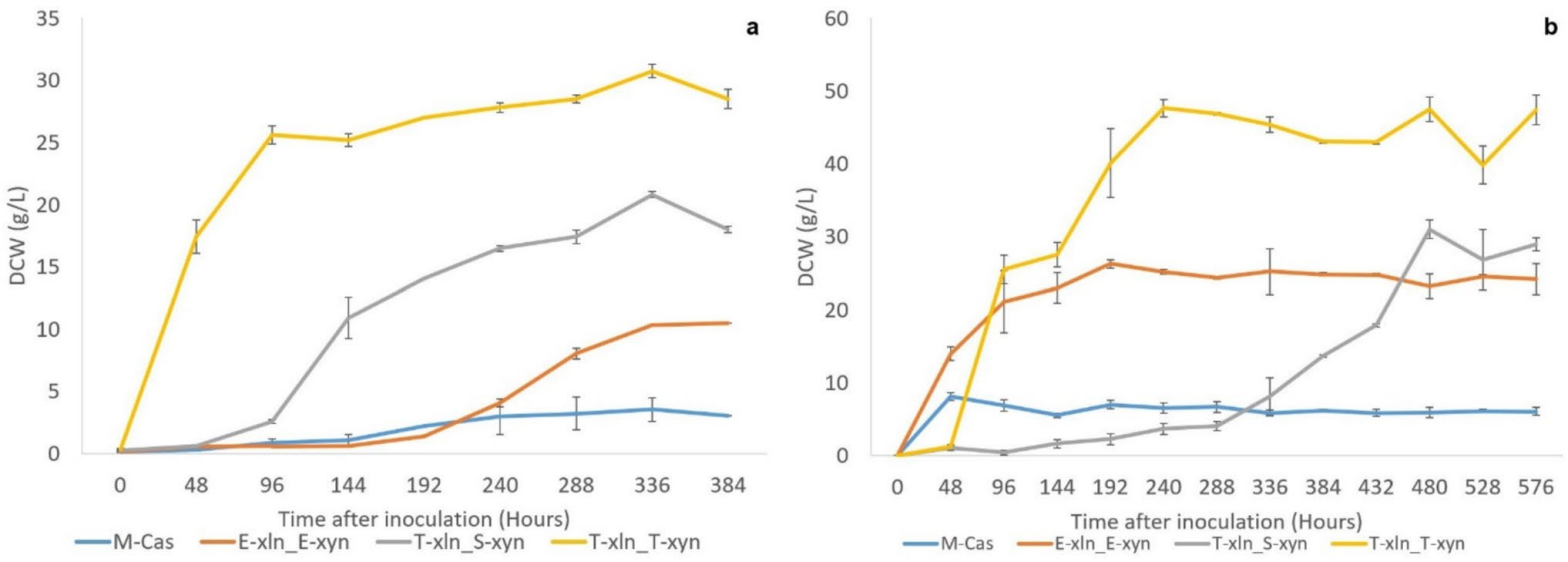
Growth trials of *S. cerevisiae* S288C-MJM121 strains engineered to contain *P. tritici-repetis* SED1-cell-tethered *xln43* and *T. reesei xyn2* under different promoter and terminator combinations. Growth (DCW g/L) on **a** XOS-containing minimal media for 16 days (384 h) and on **b** beechwood xylan-containing minimal media for 24 days (576 h). Data represents the means of triplicate cultures, with error bars indicating standard deviation

Engineered yeast strains were grown in minimal SC media containing 50 g/L XOS over a period of 16 days (384 h) with OD_600_ measurements taken at regular intervals. While the M-Cas strain contained no heterologous xylosidase or xylanase genes to aid in the degradation of XOS in the growth medium, this strain still displayed very low levels of growth likely due to the small amounts of free xylose present in the XOS substrate. The T-xln_S-xyn and T-xln_T-xyn strains showed significantly improved growth compared to the E-xln_E-xyn control strain, with T-xln_T-xyn clearly displaying the best growth overall throughout the 16-day (384 h) period (Fig. 4a). The T-xln_T-xyn strain had the shortest lag phase and reached its stationary phase after 72 h. The T-xln_S-xyn strain displayed a slightly longer lag phase, and the lag phase of the E-xln_E-xyn control strain was longer still, with these strains reaching their stationary phase after 216 and 288 h, respectively.

Yeast strains were cultivated in media containing 50 g/L beechwood xylan over 24 days (576 h) with measurements taken at regular intervals via cell counting. The parental M-Cas strain showed poor growth in xylan-containing media, as expected, as this strain contained no xylanolytic enzymes. The remaining three strains showed a spike in growth after 24–48 h of cultivation, except for the T-xln_S-xyn strain which notably displayed a lengthy lag phase, only entering its exponential phase after 312 h of cultivation, after which it was able to out-grow the benchmark E-xln_E-xyn strain (Fig. 4b). Overall, the T-xln_T-xyn strain showed the best growth when cultivated on xylan.

A phenol–sulfuric acid assay was done on the remaining cell culture upon completion of the XOS and xylan growth trials to determine quantities of remaining sugars in the growth media. No significant differences in the remaining sugar of the E-xln_E-xyn, T-xln_S-xyn, and T-xln_T-xyn cultures were observed (result not shown). The control strain expectedly showed higher amounts of residual sugar after growth in XOS and xylan media as this strain could not hydrolyse either substrate during the growth trials.

### Xylan fermentation

With pentose sugars comprising up to 30% of lignocellulosic sugars, second only to glucose, it is essential that CBP-associated *S. cerevisiae* strains are modified to contain a heterologous xylose-metabolizing pathway to ferment xylose to ethanol (Moysés et al. 2016). Xylanolytic xylose-utilizing strains such as those described here can allow CBP of xylan-containing substrates to ethanol, which can provide an economic benefit to 2G biofuel production. The engineered strains were cultivated in YP media containing 40 g/L beechwood xylan under fermentative conditions for 144 h, with samples taken at 0-, 72-, 120-, and 144-h time points, and ethanol production quantified using HPLC.

**Fig. 5:**
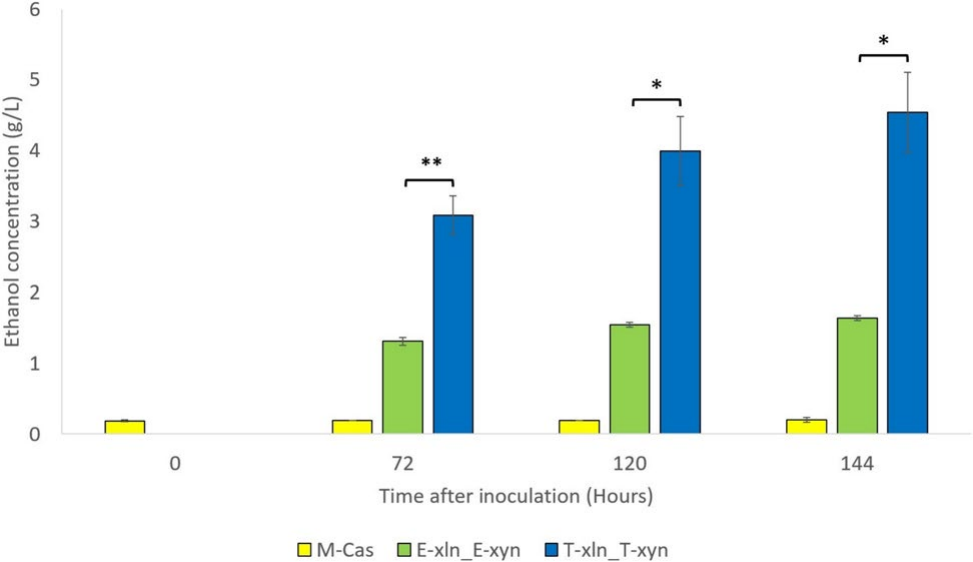
Ethanol concentrations (g/L) produced by engineered strains cultivated on YP media containing 40 g/L beechwood xylan under fermentative conditions for 144 h. Data represents the means of triplicate cultures, with error bars indicating standard deviation. Asterisks are used to indicate *p*-values calculated using the unpaired t-test assuming unequal variance: *p < 0.05; **p < 0.01; ***p < 0.001; and ****p < 0.0001

The M-Cas strain contained no heterologous xylan-hydrolysing enzymes; hence, the negligible amounts of ethanol observed for this strain are likely due to residual xylose and ethanol carried over in the inoculum. When cultivated on xylan-containing media under fermentative conditions, strains produced ethanol after 72 h of fermentation, and ethanol titres increased steadily, particularly in fermentations using the T-xln_T-xyn strain, until the end of the 144-h fermentation period. T-xln_T-xyn produced significantly more ethanol than the benchmark E-xln_E-xyn strain, with strains reaching quantities of 4.5 g/L and 1.6 g/L, respectively, after 144 h of fermentation, corresponding to 26.3% and 9.3% of the theoretical ethanol yield (Fig. 5). T-xln_T-xyn also showed faster depletion of xylose when compared to the E-xln_E-xyn benchmark strain (Table S2).

## Discussion

### Enzymatic assays

Promoters are notoriously unpredictable, displaying varying degrees of expression under different experimental setups (Myburgh et al. 2023). To properly determine the potential of the *SED1* and *TDH3* promoters in combination with the *DIT1* terminator under CBP-associated conditions, their ability to enhance heterologous xylanolytic enzyme expression was assessed in the presence and absence of glucose and under fermentative conditions.

The lower xylanase and xylosidase activity displayed by heterologous strains when cultivated on xylose and under fermentative conditions compared to that on aerobic glucose (Fig. 2) was in line with the fact that glucose is *S. cerevisiae*’s preferred carbon source and that the external stress of fermentative conditions may impose a metabolic burden on the cells, hampering their heterologous protein secretory capacity (Mao et al. 2024). Even so, both tested promoters and terminator clearly outperformed the benchmark *ENO1* promoter/terminator under all three growth conditions. A study by Guirimand et al. (2019) which made use of the *SED1* promoter, secretion signal, and anchoring domain found that when cultivated in YPD media, strains containing this “SSS” cassette showed 6- and 2.6-fold higher heterologous xylosidase and xylanase activities, respectively, when compared to a strain containing the same genes under the *TDH3* promoter and using the *GLUA* secretion signal and *SAG1* anchoring domain. This could suggest synergy between promoter and anchoring domain leading to improved enzyme activity which may account for the improved heterologous SED1-tethered xylosidase activity in the S-xln strain. However, in this study, strains containing *SED1*_P_ did not outperform those which made use of *TDH3*_P_. Rather, *TDH3*_P_ proved to be the clear superior promoter. Similar to the results seen in this study, Zhang et al. (2019) tested different promoters and anchoring regions on the expression of *Aspergillus niger* β-glucosidase and found that compared to the *SED1* promoter, the *TDH3* promoter resulted in significantly improved BGL activity.

Commonly, promoters of genes in the glycolytic pathway—a central metabolic pathway in yeast—are used in genetic engineering (Hubmann et al. 2014). Therefore, it is interesting that there is such a large disparity between the enzymatic activity of genes under the constitutive *ENO1* and *TDH3* promoters, considering that they both encode glycolytic enzymes (Guo et al. 2020). This further highlights the importance of testing promoter performance for their intended purpose. In addition to containing the strong *SED1* and *TDH3* promoters, engineered strains made use of the *DIT1* terminator, which may be an additional reason for the high enzymatic activity observed. *S. cerevisiae* terminators have been shown to increase the activity of upstream coding genes (Ito et al. 2016). Ito et al. (2013) tested five terminators to improve the secretory capacity of heterologous *T. reesei eg2* and found that strains containing the *DIT1*_T_ displayed the highest enzyme activity.

While *TDH3*_P_/*DIT1*_T_ was undoubtedly the best enhancer of *xln43_SED1*, *SED1*_P_/*DIT1*_T_ outperformed it in *xyn2* expression when cultivated on aerobic and micro-aerobic conditions on glucose—two of the three assessed cultivation conditions (Fig. 2b, c). Inokuma et al. (2014) made use of the *SED1* promoter in the enhancement of *Aspergillus aculeatus bgl1* and *T. reesei eg2* cultivated in glucose media which resulted in 8.4-fold higher β-glucosidase activity than the same strain using the *TDH3* promoter. On the other hand, Nambu-Nishida et al. (2018) reported that the *TDH3*_P_ displayed higher activity than *SED1*_P_ when cultivated in xylose media, aerobically and micro-aerobically. While comparisons across promoter studies should be made with caution due to variation in experimental setups, it may be that, while both *SED1*_P_/*DIT1*_T_ and *TDH3*_P_/*DIT1*_T_ are superior to *ENO1*_P/T_ across all three growth conditions, *SED1*_P_ may be better when used for the expression of *T. reesei xyn2* for glucose cultivation while *TDH3*_P_ remains preferable for xylose cultivation. A comparison of the data presented in Fig. 2 also indicates the frequently stated reporter protein specific nature of heterologous protein production.

Strains containing both xylosidase and xylanase genes displayed markedly lower enzyme activity when cultivated on YPX, compared to strains containing only one heterologous enzyme (Figs. 2 and 3). The decrease in activity during xylose cultivation may be due to these strains now experiencing a metabolic burden as they contain two heterologous genes instead of one, while also making use of the heterologous xylose isomerase pathway during cultivation on xylose. It remains an ongoing challenge to manipulate pathway expression, even with appropriate promoters, to achieve the desired product yield while simultaneously lessening metabolic burden (Myburgh et al. 2023). Furthermore, the significant decreases in both xylosidase and xylanase activities during xylose cultivation may be due to both of these enzymes being sensitive to product (xylose) inhibition (La Grange et al. 2001). The overexpression of xylan-hydrolases leads to the accumulation of xylo-oligomers and xylose which, in turn, inhibits these expressed xylanases in a negative feedback loop (Hidayatullah et al. 2020).

Use of the *TDH3* promoter (and *DIT1* terminator) for both genes yielded the highest xylosidase activity when cultivated aerobically (on glucose and xylose), but the combined use of *SED1*_P_ and *TDH3*_P_ (and *DIT1* terminator) produced xylosidase activity equal to that of the best-performing strain (T-xln_T-xyn) when cultivated under fermentative conditions (Fig. 3a, b). *S. cerevisiae* remodels its cell wall to adapt to ethanol stress by controlling its mannoprotein and glycoprotein metabolism through the upregulation of genes like *SED1* (Sahana et al. 2024). With the yeast cell experiencing the stress of fermentative conditions, *SED1*_P_ may have been upregulated in an effort to maintain cell wall stability through remodelling (Sanz et al. 2017), potentially affecting cell-tethered xylosidase activity and allowing it to reach levels comparable to the best-performing strain, T-xln_T-xyn.

Promoters are notoriously sensitive, with their ranking in expression levels being affected by a variety of factors (Myburgh et al. 2023). In co-producing xylosidase and xylanase strains, the use of *SED1*_P_/*DIT1*_T_ no longer resulted in the highest *xyn2* activity during cultivation on YPD and under fermentative conditions as it did when the strain contained *xyn2* only. Changes in promoter strength ranking may thus be influenced by factors as subtle as the presence of an additional gene cassette in the yeast strain.

The T-xln_S-xyn strain was created to address the possibility that its heterologous activity might be hindered by competition for transcription factors, as both heterologous genes were placed under the same promoter. However, any potential hindrance was not evident when assessing combined heterologous enzyme activity. In fact, the strain containing both heterologous genes under *TDH3*_P_/*DIT1*_T_ proved to be the best-performing strain with regard to xylosidase and xylanase activity (Fig. 3). This further supports the numerous studies across literature where *TDH3*_P_ is described as the best native yeast promoter (Myburgh et al. 2020) as well as the reporter protein specific effects observed when expressing different heterologous genes.

### Growth trials

After preliminary growth trials on YPD and YPX media (Fig. S1), it was deduced that the genetic engineering of strains to contain two additional gene cassettes imposed no significant additional metabolic burden on the strains when grown on fermentable sugars. During cultivation on media with XOS as the sole carbon source, the improved expression of heterologous *xln43_SED1* led to the improved degradation of XOS, which yielded free xylose to be metabolized by the yeast. This agreed with the xylosidase assays (Fig. 3a, b) which established that both the *SED1* and *TDH3* promoters and *DIT1* terminator allowed for the production of higher levels of heterologous xylosidase compared to *ENO1*_P/T_, enabling the yeast to hydrolyse more XOS to xylose, providing more monomeric sugars to be utilized for cell growth; hence, these strains grew more efficiently than the benchmark *ENO1*_P/T_ strain. The results also aligned with the xylanase production data (Fig. 3c, d) which showed that T-xln_T-xyn had significantly improved xylanase production compared to the benchmark strain. Xylanase will degrade longer XOS into shorter XOS, providing more short XOS for the xylosidase to act upon and increasing the overall amount of free xylose in the growth medium. This was in line with the study by Kruger and Den Haan (2022) where growth trials on XOS indicated that strains containing both secreted xylosidase and xylanase enzymes performed better than strains containing secreted xylosidase only. Therefore, the presence of a xylanase gene positively affected the growth of strains cultivated in media containing XOS. As the strains producing xylanase using the *TDH3*_P_/*DIT1*_T_ only performed the best when cultivated on xylose, it stands to reason that strains containing both genes under *TDH3*_P_/*DIT1*_T_ would display the best growth in the XOS cultivations.

While both T-xln_S-xyn and T-xln_T-xyn, once again, showed superiority over E-xln_E-xyn during growth trials on xylan, the growth pattern displayed by T-xln_S-xyn was the most unique (Fig. 4b). The breakdown of xylan requires the action of xylanase to degrade the backbone of the substrate into XOS before the xylosidase cleaves the XOS to xylose (Cunha et al. 2020). In the T-xln_S-xyn strain, the heterologous *xyn2* was cloned under *SED1*_P_, the promoter of a major stress-induced glycolytic cell wall protein. The xylanase enzyme may have only been activated in this strain on the polymeric substrate once the yeast cell was under significant stress after the small amount of free sugars in the growth medium had been depleted, which may account for the unique delay in growth of T-xln_S-xyn observed during this growth trial. One of the best described mechanisms of stress response is that caused by the depletion of available nutrients, leading to the activation of stress-responsive element (STRE) controlled genes. The *SED1* promoter contains both STRE sequences (CCCCT and AGGGG), explaining its activation during nutrient starvation (Shimoi et al. 1998) during the latter portion of the growth trial.

With T-xln_S-xyn outgrowing the *ENO1*_P/T_ benchmark strain and T-xln_T-xyn further outgrowing T-xln_S-xyn by the end of the growth trial, the superiority of these promoters and terminator during cultivations on non-native substrates and in the absence of glucose was, once again, highlighted.

### Xylan fermentation

As T-xln_T-xyn was the superior strain with regards to heterologous enzymatic activities and growth on polymeric substrates, this strain was tested in the direct fermentation of beechwood xylan to ethanol. The improved titres of ethanol produced by T-xln_T-xyn compared to the E-xln_E-xyn strain further demonstrated that the improved heterologous enzyme production, driven by our selected promoter and terminator, enhanced xylose production from xylan, facilitating greater fermentation of xylose into ethanol.

Our results of *TDH3*_P_ being the strongest tested promoter are supported by a study by Xiong et al. (2018) who tested various promoters in the presence of xylose and found that *TDH3*_P_ produced stable activity on glucose across a 72-h time period, and was the best-performing promoter when cultivated on xylose. Moreover, this promoter maintained the highest strength throughout the ethanol fermentation process, and under stressful conditions associated with fermentation (39 °C, 3.6 g/L acetic acid, and 1.0 g/L furfural). *TDH3*_P_ is clearly a superior promoter when it comes to cultivations in the absence of glucose as well as under stressful, fermentation-associated conditions, and is a viable and recommendable choice in the implementation of promoters for improved heterologous gene expression.

While the results obtained in this study are a step in the right direction of enabling CBP of LCB substrates, it also highlights certain areas that require improvement. Up to 3.77 g/L xylose remained after the 144-h fermentation period, suggesting that the M-Cas background strain was not an efficient xylose utilizer. Substrate conversion and ethanol titres can be increased through the use of a different *S. cerevisiae* strain, such as a natural or industrial strain more suited to fermentation, as opposed to the laboratory strain used in this study. Cunha et al. (2020) made use of the industrial Ethanol Red strain, engineering it to contain xylose-metabolizing pathways and heterologous β-glucosidase, β-xylosidase, and endo-β-xylanase enzymes, and achieved an ethanol titre of 11.1 g/L through the fermentation of non-detoxified hemicellulosic liquor obtained from hydrothermally pre-treated corn cob. Other studies have further reported that natural *S. cerevisiae* strains displayed greater tolerance to temperature and ethanol, and have an increased fermentation capacity in the presence of inhibitory compounds compared to industrial strains (Favaro et al. 2013, 2019). GH11 xylanases like the *T. reesei xyn2* used in this study contain large active sites and prefer cleaving main chains in unsubstituted regions (Moreira and Filho 2016). As the beechwood xylan used in our study is a glucuronoxylan, the addition of an α-glucuronidase enzyme to the engineered yeast strains could further improve their utilization of the substrate through the removal of glucuronic acid substituents on the main xylan backbone, increasing access to the xylan backbone for which the xylanase can act upon (Mert et al. 2016). However, the promoters used for heterologous expression would have to be re-assessed if any of these changes are implemented.

The results from this study show that both the *SED1*_P_/*DIT1*_T_ and *TDH3*_P_/*DIT1*_T_ promoter/terminator combinations were capable of significantly outperforming *ENO1*_P/T_ with regard to expression of the chosen xylosidase and xylanase enzymes under the assessed conditions. The *TDH3* promoter was proven to be the most reliable promoter in a variety of conditions. This held true for both single-gene expression—with one exception—and for cases where *TDH3*_P_ was used to drive the expression of two different genes in the same strain. Notably, there were no obvious signs of hindrance due to the reuse of the same transcription factors for identical gene cassettes. The T-xln_T-xyn strain also produced substantially higher amounts of ethanol when cultivated micro-aerobically on beechwood xylan compared to the benchmark E-xln_E-xyn strain, further highlighting the superiority of this promoter in a CBP process.

We have demonstrated that heterologous metabolic pathways and CBP can be significantly enhanced by employing carefully selected promoters tailored to specific conditions. The results observed from these endogenous promoters are indeed promising, but the application of promoters in industrial CBP may require further improvement. The development of hybrid and synthetic promoters is becoming an increasingly common practice in attempts to further strengthen already strong endogenous promoters to optimize protein expression in yeast. These advancements in synthetic biology present an auspicious avenue for further exploration in the enhancement of industrial CBP.

## Supplementary Information

Below is the link to the electronic supplementary material.

ESM 1 (PDF 237 KB)

## Data Availability

All data generated or analysed during this study are included in this published article and its supplementary information files.

## References

[CR1] Bailey MJ, Biely P, Poutanen K (1992) Interlaboratory testing of methods for assay of xylanase activity. J Biotechnol 23(3):257–270. 10.1016/0168-1656(92)90074-J

[CR2] Balan V (2014) Current challenges in commercially producing biofuels from lignocellulosic biomass. Int Sch Res Not. 10.1155/2014/463074 10.1155/2014/463074 PMC4393053 25937989

[CR3] Cedras G, Kroukamp H, Van Zyl WH, Den Haan R (2020) The *in vivo* detection and measurement of the unfolded protein response in recombinant cellulase producing *Saccharomyces cerevisiae* strains. Biotechnol Appl Biochem 67(1):82–94. 10.1002/bab.1819 31523843 10.1002/bab.1819

[CR4] Claes A, Deparis Q, Foulquié-Moreno MR, Thevelein JM (2020) Simultaneous secretion of seven lignocellulolytic enzymes by an industrial second-generation yeast strain enables efficient ethanol production from multiple polymeric substrates. Metab Eng 59:131–141. 10.1016/j.ymben.2020.02.004 32114024 10.1016/j.ymben.2020.02.004

[CR5] Cunha JT, Soares PO, Romaní A, Thevelein JM, Domingues L (2019) Xylose fermentation efficiency of industrial *Saccharomyces cerevisiae* yeast with separate or combined xylose reductase/xylitol dehydrogenase and xylose isomerase pathways. Biotechnol Biofuels 12:1–14. 10.1186/s13068-019-1360-8 30705706 10.1186/s13068-019-1360-8 PMC6348659

[CR6] Cunha JT, Romaní A, Inokuma K, Johansson B, Hasunuma T, Kondo A, Domingues L (2020) Consolidated bioprocessing of corn cob-derived hemicellulose: engineered industrial *Saccharomyces cerevisiae* as efficient whole cell biocatalysts. Biotechnol Biofuels 13:1–15. 10.1186/s13068-020-01780-2 32782474 10.1186/s13068-020-01780-2 PMC7414751

[CR7] Davison SA, Keller NT, van Zyl WH, den Haan R (2019) Improved cellulase expression in diploid yeast strains enhanced consolidated bioprocessing of pretreated corn residues. Enzyme Microb Technol 131:109382. 10.1016/j.enzmictec.2019.109382 31615681 10.1016/j.enzmictec.2019.109382

[CR8] Den Haan R, Kroukamp H, Mert M, Bloom M, Görgens JF, Van Zyl WH (2013) Engineering *Saccharomyces cerevisiae* for next generation ethanol production. J Chem Technol Biotechnol 88(6):983–991. 10.1002/jctb.4068

[CR9] Den Haan R, Rose SH, Cripwell RA, Trollope KM, Myburgh MW, Viljoen-Bloom M, van Zyl WH (2021) Heterologous production of cellulose-and starch-degrading hydrolases to expand *Saccharomyces cerevisiae* substrate utilization: lessons learnt. Biotechnol Adv 53:107859. 10.1016/j.biotechadv.2021.107859 34678441 10.1016/j.biotechadv.2021.107859

[CR10] Favaro L, Basaglia M, Trento A, Van Rensburg E, García-Aparicio M, Van Zyl WH, Casella S (2013) Exploring grape marc as trove for new thermotolerant and inhibitor-tolerant *Saccharomyces cerevisiae* strains for second-generation bioethanol production. Biotechnol Biofuels 6:1–14. 10.1186/1754-6834-6-168 24286305 10.1186/1754-6834-6-168 PMC4176503

[CR11] Favaro L, Jansen T, van Zyl WH (2019) Exploring industrial and natural *Saccharomyces cerevisiae* strains for the bio-based economy from biomass: the case of bioethanol. Crit Rev Biotechnol 39(6):800–816. 10.1080/07388551.2019.1619157 31230476 10.1080/07388551.2019.1619157

[CR12] Guirimand G, Inokuma K, Bamba T, Matsuda M, Morita K, Sasaki K, Ogino C, Berrin J-G, Hasunuma T, Kondo A (2019) Cell-surface display technology and metabolic engineering of *Saccharomyces cerevisiae* for enhancing xylitol production from woody biomass. Green Chem 21(7):1795–1808. 10.1039/C8GC03864C

[CR13] Guo J, Feng S, Cheng X (2020) Activity evaluation of glycolytic promoters from *Escherichia coli* and application for mevalonate biosynthesis. J Microbiol Methods 174:105946. 10.1016/j.mimet.2020.105946 32413369 10.1016/j.mimet.2020.105946

[CR14] He S, Zhang Z, Lu W (2023) Natural promoters and promoter engineering strategies for metabolic regulation in *Saccharomyces cerevisiae*. J Ind Microbiol Biotechnol 50(1):kuac029. 10.1093/jimb/kuac029 36633543 10.1093/jimb/kuac029 PMC9936215

[CR15] Hidayatullah IM, Setiadi T, Kresnowati MTAP, Boopathy R (2020) Xylanase inhibition by the derivatives of lignocellulosic material. Biores Technol 300:122740. 10.1016/j.biortech.2020.122740 10.1016/j.biortech.2020.122740 31952895

[CR16] Hubmann G, Thevelein JM, Nevoigt E (2014) Natural and modified promoters for tailored metabolic engineering of the yeast *Saccharomyces cerevisiae*. Yeast metabolic engineering: methods and protocols, pp 17–42. 10.1007/978-1-4939-0563-8_2 10.1007/978-1-4939-0563-8_2 24744025

[CR17] Inokuma K, Hasunuma T, Kondo A (2014) Efficient yeast cell-surface display of exo-and endo-cellulase using the *SED1* anchoring region and its original promoter. Biotechnol Biofuels 7(1):1–11. 10.1186/1754-6834-7-8 24423072 10.1186/1754-6834-7-8 PMC3900695

[CR18] Ito Y, Yamanishi M, Ikeuchi A, Imamura C, Tokuhiro K, Kitagawa T, Matsuyama T (2013) Characterization of five terminator regions that increase the protein yield of a transgene in *Saccharomyces cerevisiae*. J Biotechnol 168(4):486–492. 10.1016/j.jbiotec.2013.09.024 24126155 10.1016/j.jbiotec.2013.09.024

[CR19] Ito Y, Kitagawa T, Yamanishi M, Katahira S, Izawa S, Irie K, Furutani-Seiki M, Matsuyama T (2016) Enhancement of protein production via the strong *DIT1* terminator and two RNA-binding proteins in *Saccharomyces cerevisiae*. Sci Rep 6(1):36997. 10.1038/srep36997 27845367 10.1038/srep36997 PMC5109538

[CR20] Jacob O, van Lill GR, den Haan R (2022) CRISPR-based multi-gene integration strategies to create *Saccharomyces cerevisiae* strains for consolidated bioprocessing. Appl Sci 12(23):12317. 10.3390/app122312317

[CR21] Jansen ML, Bracher JM, Papapetridis I, Verhoeven MD, de Bruijn H, de Waal PP, van Maris AJ, Klaassen P, Pronk JT (2017) *Saccharomyces cerevisiae* strains for second-generation ethanol production: from academic exploration to industrial implementation. FEMS Yeast Res 17(5). 10.1093/femsyr/fox044 10.1093/femsyr/fox044 PMC5812533 28899031

[CR22] Kroukamp H, den Haan R, van Zyl JH, van Zyl WH (2018) Rational strain engineering interventions to enhance cellulase secretion by *Saccharomyces cerevisiae*. Biofuels, Bioprod Biorefin 12(1):108–124. 10.1002/bbb.1824

[CR23] Kruger F, Den Haan R (2022) Surface tethered xylosidase activity improved xylan conversion in engineered strains of *Saccharomyces cerevisiae*. J Chem Technol Biotechnol 97(5):1099–1111. 10.1002/jctb.7044

[CR24] La Grange DC, Pretorius IS, Claeyssens M, Van Zyl WH (2001) Degradation of xylan to D-xylose by recombinant *Saccharomyces cerevisiae* coexpressing the *Aspergillus niger β-xylosidase (xlnD)* and the *Trichoderma reesei xylanase II (xyn2)* genes. Appl Environ Microbiol 67(12):5512–5519. 10.1128/AEM.67.12.5512-5519.2001 11722900 10.1128/AEM.67.12.5512-5519.2001 PMC93337

[CR25] Lane S, Dong J, Jin Y-S (2018) Value-added biotransformation of cellulosic sugars by engineered *Saccharomyces cerevisiae*. Biores Technol 260:380–394. 10.1016/j.biortech.2018.04.013 10.1016/j.biortech.2018.04.013 29655899

[CR26] Li X, Wang Z, Zhang G, Yi L (2019) Improving lycopene production in *Saccharomyces cerevisiae* through optimizing pathway and chassis metabolism. Chem Eng Sci 193:364–369. 10.1016/j.ces.2018.09.030

[CR27] Lugani Y, Rai R, Prabhu AA, Maan P, Hans M, Kumar V, Kumar S, Chandel AK, Sengar R (2020) Recent advances in bioethanol production from lignocelluloses: a comprehensive review with a focus on enzyme engineering and designer biocatalysts. Biofuel Res J 7(4):1267–1295. 10.18331/BRJ2020.7.4.5

[CR28] Mao J, Zhang H, Chen Y, Wei L, Liu J, Nielsen J, Chen Y, Xu N (2024) Relieving metabolic burden to improve robustness and bioproduction by industrial microorganisms. Biotechnol Adv :108401. 10.1016/j.biotechadv.2024.108401 10.1016/j.biotechadv.2024.108401 38944217

[CR29] Mert MJ, La Grange DC, Rose SH, van Zyl WH (2016) Engineering of *Saccharomyces cerevisiae* to utilize xylan as a sole carbohydrate source by co-expression of an endoxylanase, xylosidase and a bacterial xylose isomerase. J Ind Microbiol Biotechnol 43(4):431–440. 10.1007/s10295-015-1727-1 26749525 10.1007/s10295-015-1727-1

[CR30] Mert M, Rose S, La Grange D, Bamba T, Hasunuma T, Kondo A, van Zyl W (2017) Quantitative metabolomics of a xylose-utilizing *Saccharomyces cerevisiae* strain expressing the *Bacteroides thetaiotaomicron* xylose isomerase on glucose and xylose. J Ind Microbiol Biotechnol 44(10):1459–1470. 10.1007/s10295-017-1969-1 28744577 10.1007/s10295-017-1969-1

[CR31] Mikkelsen MD, Buron LD, Salomonsen B, Olsen CE, Hansen BG, Mortensen UH, Halkier BA (2012) Microbial production of indolylglucosinolate through engineering of a multi-gene pathway in a versatile yeast expression platform. Metab Eng 14(2):104–111 22326477 10.1016/j.ymben.2012.01.006

[CR32] Moreira L, Filho E (2016) Insights into the mechanism of enzymatic hydrolysis of xylan. Appl Microbiol Biotechnol 100:5205–5214. 10.1007/s00253-016-7555-z 27112349 10.1007/s00253-016-7555-z

[CR33] Moysés DN, Reis VCB, Almeida JRMd, Moraes LMPd, Torres FAG (2016) Xylose fermentation by *Saccharomyces cerevisiae*: challenges and prospects. Int J Mol Sci 17(3):207. 10.3390/ijms17030207 26927067 10.3390/ijms17030207 PMC4813126

[CR34] Mujtaba M, Fraceto LF, Fazeli M, Mukherjee S, Savassa SM, de Medeiros GA, Santo Pereira AdE, Mancini SD, Lipponen J, Vilaplana F (2023) Lignocellulosic biomass from agricultural waste to the circular economy: a review with focus on biofuels, biocomposites and bioplastics. J Clean Prod 402:136815. 10.1016/j.jclepro.2023.136815

[CR35] Myburgh MW, Rose SH, Viljoen-Bloom M (2020) Evaluating and engineering *Saccharomyces cerevisiae* promoters for increased amylase expression and bioethanol production from raw starch. FEMS Yeast Res 20(6). 10.1093/femsyr/foaa047 10.1093/femsyr/foaa047 32785598

[CR36] Myburgh MW, Schwerdtfeger KS, Cripwell RA, van Zyl WH, Viljoen-Bloom M (2023) Promoters and introns as key drivers for enhanced gene expression in *Saccharomyces cerevisiae.* Advances in Applied Microbiology, vol 124. Elsevier, pp 1–29 10.1016/bs.aambs.2023.07.002 37597945

[CR37] Nambu-Nishida Y, Sakihama Y, Ishii J, Hasunuma T, Kondo A (2018) Selection of yeast *Saccharomyces cerevisiae* promoters available for xylose cultivation and fermentation. J Biosci Bioeng 125(1):76–86. 10.1016/j.jbiosc.2017.08.001 28869192 10.1016/j.jbiosc.2017.08.001

[CR38] Oh EJ, Jin Y-S (2020) Engineering of *Saccharomyces cerevisiae* for efficient fermentation of cellulose. FEMS Yeast Res 20(1):foz089. 10.1093/femsyr/foz089 31917414 10.1093/femsyr/foz089

[CR39] Olguin-Maciel E, Singh A, Chable-Villacis R, Tapia-Tussell R, Ruiz HA (2020) Consolidated bioprocessing, an innovative strategy towards sustainability for biofuels production from crop residues: an overview. Agronomy 10(11):1834. 10.3390/agronomy10111834

[CR40] Partow S, Siewers V, Bjørn S, Nielsen J, Maury J (2010) Characterization of different promoters for designing a new expression vector in *Saccharomyces cerevisiae*. Yeast 27(11):955–964. 10.1002/yea.1806 20625983 10.1002/yea.1806

[CR41] Peng B, Williams TC, Henry M, Nielsen LK, Vickers CE (2015) Controlling heterologous gene expression in yeast cell factories on different carbon substrates and across the diauxic shift: a comparison of yeast promoter activities. Microb Cell Fact 14(1):1–11. 10.1186/s12934-015-0278-5 26112740 10.1186/s12934-015-0278-5 PMC4480987

[CR42] Sahana GR, Balasubramanian B, Joseph KS, Pappuswamy M, Liu W-C, Meyyazhagan A, Kamyab H, Chelliapan S, Joseph BV (2024) A review on ethanol tolerance mechanisms in yeast: Current knowledge in biotechnological applications and future directions. Process Biochem 138:1–13. 10.1016/j.procbio.2023.12.024

[CR43] Sakamoto T, Hasunuma T, Hori Y, Yamada R, Kondo A (2012) Direct ethanol production from hemicellulosic materials of rice straw by use of an engineered yeast strain codisplaying three types of hemicellulolytic enzymes on the surface of xylose-utilizing *Saccharomyces cerevisiae* cells. J Biotechnol 158(4):203–210. 10.1016/j.jbiotec.2011.06.025 21741417 10.1016/j.jbiotec.2011.06.025

[CR44] Sanz AB, García R, Rodríguez-Peña JM, Arroyo J (2017) The CWI pathway: regulation of the transcriptional adaptive response to cell wall stress in yeast. Journal of Fungi 4(1):1. 10.3390/jof4010001 29371494 10.3390/jof4010001 PMC5872304

[CR45] Sharma J, Kumar V, Prasad R, Gaur NA (2022) Engineering of *Saccharomyces cerevisiae* as a consolidated bioprocessing host to produce cellulosic ethanol: Recent advancements and current challenges. Biotechnol Adv 56:107925. 10.1016/j.biotechadv.2022.107925 35151789 10.1016/j.biotechadv.2022.107925

[CR46] Shimoi H, Kitagaki H, Ohmori H, Iimura Y, Ito K (1998) Sed1p is a major cell wall protein of Saccharomyces cerevisiae in the stationary phase and is involved in lytic enzyme resistance. J Bacteriol 180(13):3381–3387. 10.1128/jb.180.13.3381-3387.1998 9642191 10.1128/jb.180.13.3381-3387.1998 PMC107293

[CR47] Sun J, Shao Z, Zhao H, Nair N, Wen F, Xu JH, Zhao H (2012) Cloning and characterization of a panel of constitutive promoters for applications in pathway engineering in *Saccharomyces cerevisiae*. Biotechnol Bioeng 109(8):2082–2092. 10.1002/bit.24481 22383307 10.1002/bit.24481

[CR48] Tang H, Wu Y, Deng J, Chen N, Zheng Z, Wei Y, Luo X, Keasling JD (2020) Promoter architecture and promoter engineering in *Saccharomyces cerevisiae*. Metabolites 10(8):320. 10.3390/metabo10080320 32781665 10.3390/metabo10080320 PMC7466126

[CR49] Thomson K, Bhat A, Carvell J (2015) Comparison of a new digital imaging technique for yeast cell counting and viability assessments with traditional methods. J Inst Brew 121(2):231–237. 10.1002/jib.224

[CR50] Thuring R, Sanders J, Borst P (1975) A freeze-squeeze method for recovering long DNA from agarose gels. Anal Biochem 66(1):213–220. 10.1016/0003-2697(75)90739-3 1096670 10.1016/0003-2697(75)90739-3

[CR51] Valenzuela-Ortega M, French CE (2019) Engineering of industrially important microorganisms for assimilation of cellulosic biomass: towards consolidated bioprocessing. Biochem Soc Trans 47(6):1781–1794. 10.1042/BST20190293 31845725 10.1042/BST20190293

[CR52] Van Dyk J, Pletschke B (2012) A review of lignocellulose bioconversion using enzymatic hydrolysis and synergistic cooperation between enzymes—factors affecting enzymes, conversion and synergy. Biotechnol Adv 30(6):1458–1480. 10.1016/j.biotechadv.2012.03.002 22445788 10.1016/j.biotechadv.2012.03.002

[CR53] Weinhandl K, Winkler M, Glieder A, Camattari A (2014) Carbon source dependent promoters in yeasts. Microb Cell Fact 13(1):1–17. 10.1186/1475-2859-13-5 24401081 10.1186/1475-2859-13-5 PMC3897899

[CR54] Xiao W, Li H, Xia W, Yang Y, Hu P, Zhou S, Hu Y, Liu X, Dai Y, Jiang Z (2019) Co-expression of cellulase and xylanase genes in *Saccharomyces cerevisiae* toward enhanced bioethanol production from corn stover. Bioengineered 10(1):513–521. 10.1080/21655979.2019.1682213 31661645 10.1080/21655979.2019.1682213 PMC6844370

[CR55] Xiong L, Zeng Y, Tang R-Q, Alper HS, Bai F-W, Zhao X-Q (2018) Condition-specific promoter activities in *Saccharomyces cerevisiae*. Microb Cell Fact 17(1):1–15. 10.1186/s12934-018-0899-6 29631591 10.1186/s12934-018-0899-6 PMC5891911

[CR56] Xu N, Wei L, Liu J (2019) Recent advances in the applications of promoter engineering for the optimization of metabolite biosynthesis. World J Microbiol Biotechnol 35:1–10. 10.1007/s11274-019-2606-0 10.1007/s11274-019-2606-0 30706208

[CR57] Yang S, Song L, Wang J, Zhao J, Tang H, Bao X (2023) Engineering *Saccharomyces cerevisiae* for efficient production of recombinant proteins. Eng Microbiol :100122. 10.1016/j.engmic.2023.100122 10.1016/j.engmic.2023.100122 PMC11611019 39628786

[CR58] Zhang Y, Min Z, Qin Y, Ye D-Q, Song Y-Y, Liu Y-L (2019) Efficient Display of *Aspergillus niger* β-glucosidase on *Saccharomyces cerevisiae* cell wall for aroma enhancement in wine. J Agric Food Chem 67(18):5169–5176 30997795 10.1021/acs.jafc.9b00863

