# Supplementary material for: Identifying promoters to enhance heterologous gene expression in recombinant Saccharomyces cerevisiae strains cultivated on non-native substrates
Source: Appl Microbiol Biotechnol. 2025 Jul 26;109:173. doi: 10.1007/s00253-025-13563-6 (PMC12296805; doi:10.1007/s00253-025-13563-6)
Supplement: Supplementary file 1 [file 253_2025_13563_MOESM1_ESM.pdf]

Supplementary material for:

Identifying promoters to enhance heterologous gene expression in recombinant yeast cultivated on non-native substrates

Jordan Fortuin[1] and Riaan den Haan[1*]

[1]Department of Biotechnology, University of the Western Cape, Bellville, South Africa

[*]Corresponding author: Tel no.: +27 21 959-2199 Email address: rdenhaan@uwc.ac.za

Jordan Fortuin ORCID: 0000-0001-7528-806X

Riaan den Haan ORCID: 0000-0001-6983-6226

Table S1: Yeast strains constructed in this study and their respective culture collection numbers at the publicly accessible Biobanks South Africa Yeast Culture Collection, Department of Microbiology and Biochemistry, University of the Free State. For more details, refer to table 1 in the main text.

**Strain name**	**Accession Form Nr**	**UFS Collection Nr**	**Other Nr (Strain Nr)**
SED1$_{\text{P}}$-*xln43_SED1-DIT1*$_{\text{T}}$ Ch10	4070	UOFS Y-4424	S-xln
TDH3$_{\text{P}}$-*xln43_SED1-DIT1*$_{\text{T}}$ Ch10	4071	UOFS Y-4425	T-xln
SED1$_{\text{P}}$-*xyn2-DIT1*$_{\text{T}}$ Ch11	4073	UOFS Y-4427	S-xyn
TDH3$_{\text{P}}$-*xyn2-DIT1*$_{\text{T}}$ Ch11	4072	UOFS Y-4426	T-xyn
TDH3$_{\text{P}}$-*xln43_SED1-DIT1*$_{\text{T}}$ Ch10 + *SED1*$_{\text{P}}$-*xyn2-DIT1*$_{\text{T}}$ Ch11	4074	UOFS Y-4428	T-xln_S-xyn
TDH3$_{\text{P}}$-*xln43*_SED1-*DIT1*$_{\text{T}}$ Ch10 + *TDH3*$_{\text{P}}$-*xyn2-DIT1*$_{\text{T}}$ Ch11	4975	UOFS Y-4429	T-xln_T-xyn

Table S2. Xylose concentrations (g/L) obtained by engineered strains cultivated on YP media containing 40 g/L beechwood xylan under fermentative conditions during a 144-hour time period.

	**T0**	**T72**	**T120**	**T144**
**MJM**	0.59 ± 0.02	0.0	0.0	0.0
**E-xln_E-xyn**	0.95 ± 0.06	4.16 ± 0.09	3.94 ± 0.06	3.77 ± 0.03
**T-xln_T-xyn**	0.94 ± 0.01	3.86 ± 0.25	3.09 ± 0.25	2.96 ± 0.29

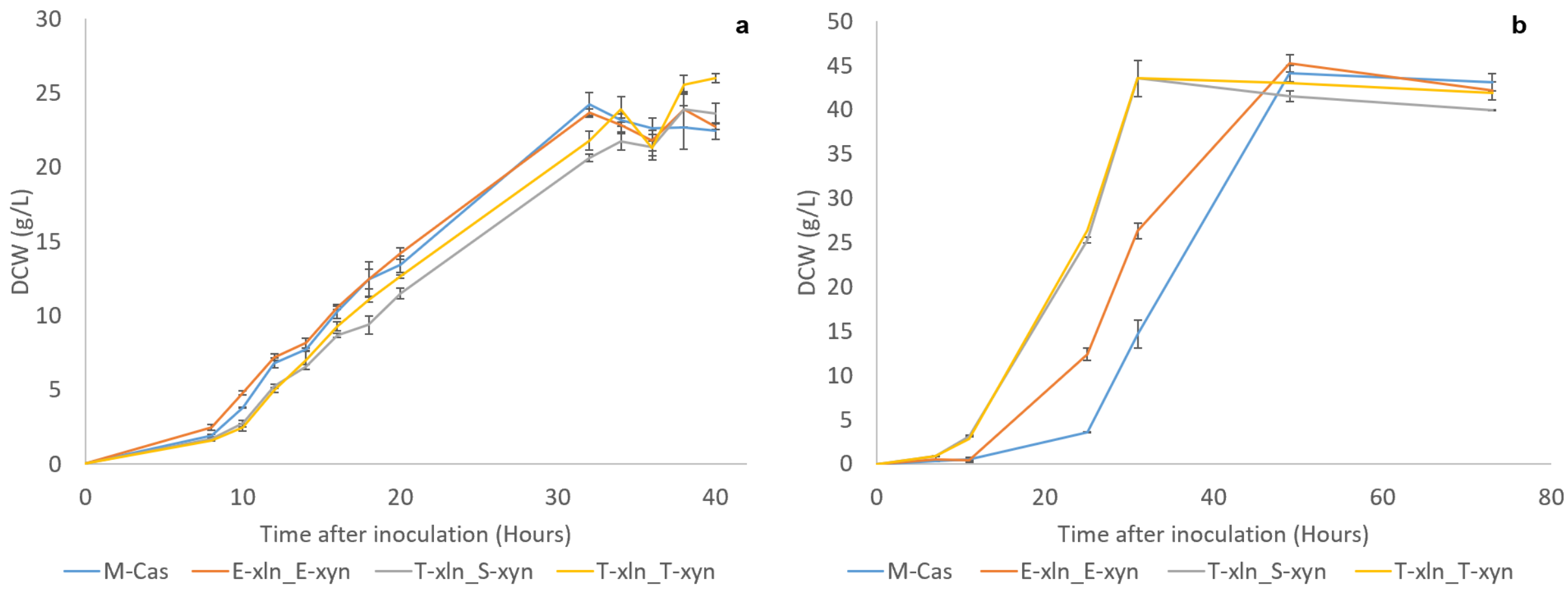

Fig. S1. Growth in DCW (g/L) of selected *S. cerevisiae* S288C strains containing xylosidase and xylanase under different promoter and terminator combinations in (a) glucose-containing media (YPD) and (b) xylose-containing media (YPX).
